# Prognostic value of the atherogenic index of plasma for early-stage diabetic kidney disease in type 2 diabetes: a retrospective cohort study using supervised machine learning

**DOI:** 10.3389/fnut.2026.1844510

**Published:** 2026-06-30

**Authors:** Yazhi Wang, Hui Chen

**Affiliations:** 1 The Second School of Clinical Medicine, Lanzhou University, Lanzhou, Gansu, China; 2 Department of Endocrinology, Lanzhou University Second Hospital, Lanzhou, Gansu, China

**Keywords:** atherogenic index of plasma, cohort study, early-stage DKD, prognostic prediction model, supervised machine learning, type 2 diabetes mellitus

## Abstract

**Background:**

Diabetic kidney disease (DKD) is the leading cause of end-stage renal disease (ESRD). Its insidious onset means that once it progresses to the stage of heavy proteinuria or significant decline in renal function, treatment difficulty and burden increase dramatically. Therefore, identifying high-risk individuals in the early-stage DKD (ES-DKD, stages 1–2) and intervening promptly is crucial for delaying disease progression and improving prognosis. The atherogenic index of plasma (AIP) is a novel composite indicator reflecting dyslipidemia and insulin resistance. However, its prognostic value in ES-DKD among patients with type 2 diabetes mellitus (T2DM) remains unclear. This study aims to investigate the association between AIP and the risk of developing ES-DKD in T2DM patients, and to develop prognostic prediction models using supervised machine learning (SML) algorithms.

**Methods:**

Clinical data of 1,006 patients with T2DM were extracted from the hospital information system. The study population was divided into three groups according to the tertiles of baseline AIP values. The study endpoint was the occurrence of ES-DKD during follow-up. Cox proportional hazards regression models and subgroup analyses were used to assess the association between AIP and the risk of ES-DKD. Restricted cubic spline (RCS) analysis was applied to examine the linear trend of the dose–response relationship. Differences between groups were compared using Kaplan–Meier survival curves and the log-rank test. The Boruta algorithm was employed to evaluate the importance of AIP among the predictor variables. Feature selection was further performed using least absolute shrinkage and selection operator (LASSO) regression and backward stepwise selection. Prognostic prediction models were constructed based on six SML algorithms, including random survival forest (RSF), LASSO-Cox, CoxBoost, extreme gradient boosting (XGBoost), supervised principal components (superpc), and partial least squares regression for the Cox model (plsRcox). Model performance was evaluated using time-dependent area under the curve (AUC), calibration curves, and decision curve analysis (DCA).

**Results:**

Among the 1,006 T2DM patients, the median follow-up time was 49 months, and 404 ES-DKD events occurred. Multivariable Cox regression analysis showed that for each one-unit increase in AIP, the risk of ES-DKD increased by 74% (HR = 1.74, 95% CI: 1.19–2.54, *p* = 0.004). Compared with the lowest AIP tertile group, patients in the highest tertile group had a 33% increased risk of ES-DKD (HR = 1.33, 95% CI: 1.01–1.74, *p* = 0.039). Subgroup analyses yielded robust results, and RCS analysis indicated a linear positive correlation between AIP and the risk of ES-DKD. Kaplan–Meier curves showed that the long-term event-free survival rate was significantly lower in the high AIP group. Boruta feature selection results demonstrated that AIP had a high importance score. Among the six SML models, the prognostic prediction model based on the XGBoost algorithm exhibited the best performance, achieving the highest time-dependent AUC. In the training and validation cohorts, the AUCs for predicting the 1-, 3-, and 5-year risk of ES-DKD ranged from 0.779 to 0.809 and 0.752 to 0.784, respectively. Calibration curves showed good agreement between predicted probabilities and observed probabilities, and DCA indicated that the model provided high clinical net benefit. To facilitate the application of ES-DKD risk prediction in clinical practice, a web-based tool was also developed in this study.

**Conclusion:**

AIP is an independent prognostic factor for the occurrence of ES-DKD in patients with T2DM. Integrating this low-cost, easily accessible biomarker into SML frameworks may provide effective tools for early risk stratification and individualized management.

## Graphical abstract

**Figure:**
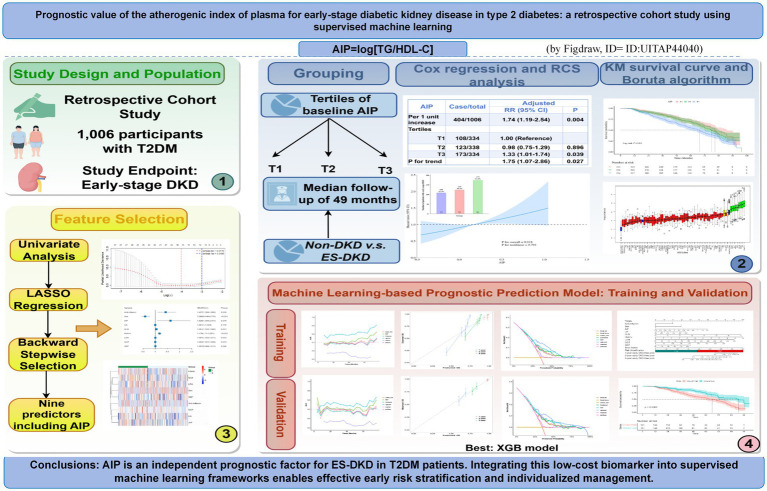
Created with Figdraw.com.

## Introduction

Diabetic kidney disease (DKD) is one of the most common and severe microvascular complications of type 2 diabetes mellitus (T2DM) and has become the leading cause of end-stage renal disease (ESRD) worldwide (1). According to the International Diabetes Federation (IDF), the global prevalence of diabetes among adults aged 20–79 years was approximately 10.5% (536.6 million) in 2021, and is projected to rise to 12.2% (783.2 million) by 2045 (2). Among these individuals, approximately 20–40% of patients with T2DM will develop DKD (3). DKD not only significantly increases the risk of cardiovascular events and all-cause mortality, but also imposes a substantial economic burden on patients, their families, and healthcare systems (4). Notably, DKD has an insidious onset, often presenting with no specific clinical symptoms in its early stages. Once it progresses to the stage of heavy proteinuria or significant decline in renal function (i.e., clinical-stage DKD), existing treatment modalities are often unable to reverse the disease, leading to a sharp increase in both treatment difficulty and healthcare burden (5). Therefore, accurately identifying high-risk individuals and implementing proactive interventions during the early stages of DKD—specifically early-stage DKD (ES-DKD; stages 1–2), characterized by normal or mildly reduced estimated glomerular filtration rate (eGFR ≥ 60 mL/min/1.73 m^2^) with the onset of increased urinary albumin excretion (urinary albumin-to-creatinine ratio, UACR ≥ 30 mg/g)—is of critical importance for delaying disease progression and improving long-term patient outcomes (6). Exploring reliable prognostic indicators represents a key approach to addressing this challenge.

Insulin resistance (IR) has also been established as an independent risk factor for the development and progression of DKD (7). It contributes to kidney injury through multiple mechanisms, including induction of endothelial dysfunction, exacerbation of oxidative stress, activation of inflammatory pathways, and promotion of glomerular mesangial cell proliferation. Additionally, hyperinsulinemia may facilitate renal fibrosis by stimulating the expression of insulin-like growth factor-1 and transforming growth factor-*β* (8, 9). The hyperinsulinemic-euglycemic clamp technique, considered the gold standard for assessing IR, is technically complex, costly, and invasive, limiting its widespread application in clinical practice (10). Therefore, identifying simple and reliable surrogate markers of IR holds significant clinical value for risk stratification of ES-DKD in the T2DM population.

The atherogenic index of plasma (AIP) is a novel composite lipid parameter that has garnered considerable attention in recent years (11, 12). This index comprehensively reflects the balance between the atherogenic lipid burden and the protective factors against atherosclerosis. It has been shown to be negatively correlated with low-density lipoprotein particle diameter and positively correlated with small dense low-density lipoprotein (sdLDL) levels, thereby enabling a more comprehensive assessment of the atherogenic potential of dyslipidemia (13). Accumulating evidence indicates that AIP is not only closely associated with cardiovascular events such as coronary artery disease (13), myocardial infarction (14), and ischemic stroke (15), but also serves as an effective surrogate marker of IR (16, 17). A cross-sectional study demonstrated that AIP was significantly positively correlated with the homeostatic model assessment of insulin resistance and exhibited superior predictive performance for IR compared with conventional individual lipid parameters (17). Furthermore, AIP has been found to be closely associated with the risk of various IR-related conditions, including metabolic syndrome (18) and metabolic dysfunction-associated steatotic liver disease (MASLD) (19).

Although AIP has been widely used to predict the aforementioned cardiometabolic diseases (CMD), research on its role in risk stratification for chronic kidney disease (CKD) in the diabetic population remains limited. Existing studies have primarily focused on clinical-stage DKD (stage 3 or above) as the endpoint and are largely cross-sectional in design, with no systematic evaluation of the prognostic value of AIP for ES-DKD (stages 1–2) (20, 21). Notably, ES-DKD represents a critical “window of opportunity” for therapeutic intervention and delaying disease progression. Moreover, previous studies have not fully leveraged the advantages of modern machine learning (ML), particularly supervised machine learning (SML) algorithms, in handling high-dimensional data and capturing nonlinear relationships (22). ML techniques can integrate multidimensional clinical data, capture complex interactions among variables, and construct more accurate risk prediction models, thereby providing robust support for clinical decision-making (22, 23). Therefore, based on real-world data and long-term follow-up results, this study aims to investigate the association between AIP and the risk of ES-DKD in patients with T2DM. Furthermore, we seek to develop and validate ES-DKD prognostic prediction models incorporating AIP using SML algorithms, with the goal of providing clinicians with additional reference information for identifying high-risk patients and guiding individualized primary prevention strategies.

## Methods

### Study design and ethical approval

This study was a retrospective observational cohort study conducted at Lanzhou University Second Hospital. Data were extracted from the hospital information system (HIS), and eligible patients were enrolled between January 1, 2017, and December 31, 2020, with subsequent follow-up. The follow-up period for outcome assessment ended in January 2026, with a median follow-up duration of 49 months. This study was approved by the Ethics Committee of Lanzhou University Second Hospital (approval No. 2022A-187). Due to the retrospective study design and the de-identification of patient information to protect privacy, the requirement for informed consent was waived. This study was conducted in accordance with the principles of the Declaration of Helsinki, the Reporting of studies Conducted using Observational Routinely-collected Data (RECORD) guideline (24), and the Transparent Reporting of a multivariable prediction model for Individual Prognosis Or Diagnosis + Artificial Intelligence (TRIPOD-AI) statement (25).

### Inclusion and exclusion criteria

**Figure 1:**
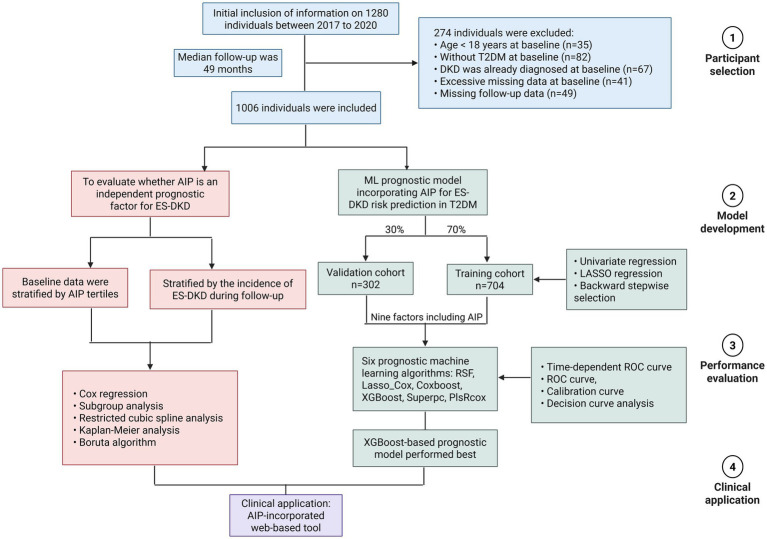
Overview of the study workflow, including participant selection, exclusion criteria, follow-up, model development, validation, and statistical analysis. AIP, atherogenic index of plasma; ES-DKD, early-stage diabetic kidney disease; T2DM, type 2 diabetes mellitus; RSF, random survival forest; Lasso_Cox, lasso-penalized cox proportional hazards model; XGBoost, extreme gradient boosting; superpc, supervised principal components; plsRcox, partial least squares regression for cox model; ROC, receiver operating characteristic.

Inclusion criteria were as follows: (1) diagnosis of T2DM at baseline (26); (2) age ≥ 18 years at baseline; (3) availability of relatively complete baseline clinical data; (4) baseline eGFR ≥ 60 mL/(min·1.73 m^2^), negative urinary protein (urinalysis), and UACR < 30 mg/g. Exclusion criteria were as follows: (1) diagnosis of type 1 diabetes mellitus (T1DM), gestational diabetes mellitus (GDM), or other specific types of diabetes at baseline; (2) presence of acute diabetic complications at baseline, such as diabetic ketoacidosis or hyperosmolar hyperglycemic coma; (3) presence of acute kidney injury, severe infection, stress-related conditions, or cancer cachexia at baseline; (4) diagnosis of DKD at baseline; (5) missing follow-up data. A total of 1,280 patients with T2DM were initially screened. Ultimately, 1,006 patients met all criteria and were included in the final analysis. A detailed flowchart of patient selection is presented in Figure 1.

### Data collection and preprocessing

This study collected comprehensive baseline clinical data from patients with T2DM, including demographic characteristics, clinical indicators, complications and comorbidities, medications, lifestyle factors and family history, biochemical parameters, hormones, and routine blood tests, comprising a total of 72 variables (see Table 1). Supplementary Table S1 presents the missing rates for each variable, all of which were less than 10%. Therefore, missing values were imputed using the random forest imputation method. One-hot encoding was applied to categorical variables without inherent order or hierarchical relationships by creating separate binary features for each category. For example, the variable sex, with categories “male” or “female,” was transformed into a binary variable indicating “whether male” (27). Label encoding was applied to categorical variables with a clear order or hierarchical relationship (i.e., ordinal variables) by assigning a unique integer value to each category. For instance, smoking status (yes or no) was converted to 1 and 0, respectively (27).

**Table 1:** Baseline characteristics of the study population across atherogenic index of plasma tertiles.

Characteristics	Overall	T1	T2	T3	*P*-value
	(*n* = 1,006)	(*n* = 334)	(*n* = 338)	(*n* = 334)	
Demographics					
Age (years)	56.53 ± 9.99	59.44 ± 8.80	57.08 ± 9.54	53.05 ± 10.50	**<0.001**
Male, *n* (%)	651 (64.71)	191 (57.19)	220 (65.09)	240 (71.86)	**<0.001**
Ethnicity-Han, *n* (%)	954 (94.83)	319 (95.51)	319 (94.38)	316 (94.61)	0.784
Married, *n* (%)	979 (97.32)	323 (96.71)	329 (97.34)	327 (97.90)	0.632
Career-Business/Service, *n* (%)	704 (69.98)	251 (75.15)	226 (66.86)	227 (67.96)	0.062
Comorbidities					
HTN, *n* (%)	334 (33.20)	130 (38.92)	107 (31.66)	97 (29.04)	**0.019**
MASLD, *n* (%)	486 (48.31)	106 (31.74)	163 (48.22)	217 (64.97)	**<0.001**
CVD, *n* (%)	630 (62.62)	226 (67.66)	217 (64.20)	187 (55.99)	**0.006**
DR, *n* (%)	502 (49.90)	190 (56.89)	168 (49.70)	144 (43.11)	**0.002**
DSPN, *n* (%)	823 (81.81)	287 (85.93)	267 (78.99)	269 (80.54)	0.051
DPVD, *n* (%)	270 (26.84)	93 (27.84)	96 (28.40)	81 (24.25)	0.421
Lifestyle and FM					
Smoking, *n* (%)	293 (29.13)	86 (25.75)	96 (28.40)	111 (33.23)	0.097
Drinking, *n* (%)	220 (21.87)	67 (20.06)	73 (21.60)	80 (23.95)	0.472
FM of DM, *n* (%)	348 (34.59)	118 (35.33)	114 (33.73)	116 (34.73)	0.907
FM of HTN, *n* (%)	156 (15.51)	51 (15.27)	47 (13.91)	58 (17.37)	0.459
Medications					
Insulins, *n* (%)	473 (47.02)	146 (43.71)	159 (47.04)	168 (50.30)	0.234
NIAD, *n* (%)	991 (98.51)	328 (98.20)	332 (98.22)	331 (99.10)	0.585
LLA, *n* (%)	486 (48.31)	127 (38.02)	143 (42.31)	216 (64.67)	**<0.001**
Anti-inflamm, *n* (%)	252 (25.05)	90 (26.95)	84 (24.85)	78 (23.35)	0.560
Renoprot, *n* (%)	24 (2.39)	10 (2.99)	6 (1.78)	8 (2.40)	0.585
AH, *n* (%)	328 (32.60)	128 (38.32)	111 (32.84)	89 (26.65)	**0.006**
Clinical indicators					
Course (years)	7.00 (3.00, 12.00)	8.00 (5.00,13.00)	8.00 (2.00,12.00)	6.00 (2.00,10.00)	**<0.001**
SBP (mmHg)	131.00 (121.00, 142.00)	131.00 (121.00,145.00)	131.00 (122.00,142.00)	130.00 (121.00,141.00)	0.680
DBP (mmHg)	78.00 (73.00, 85.00)	77.00 (71.00,83.00)	77.00 (73.00,85.00)	79.00 (74.00,86.00)	**<0.001**
BMI (kg/m2)	24.22 (22.31, 26.16)	23.52 (21.48,25.30)	24.38 (22.47,25.90)	24.92 (23.02,27.03)	**<0.001**
Biochemical indicators					
Glucose metabolic indices					
HbA1c (%)	7.90 (6.80, 9.60)	7.60 (6.70,9.07)	8.00 (6.90,9.60)	8.30 (7.00,10.00)	**<0.001**
FBG (mmo/L)	8.50 (7.10, 10.80)	8.10 (6.90,9.80)	8.40 (7.00,10.47)	9.30 (7.50,11.70)	**<0.001**
FINS (mU/L)	7.33 (5.09, 10.40)	6.21 (3.93,9.55)	7.50 (5.26,10.58)	8.27 (5.92,11.54)	**<0.001**
FCP (ng/mL)	1.42 (1.05, 1.84)	1.20 (0.82,1.64)	1.42 (1.08,1.79)	1.66 (1.27,2.15)	**<0.001**
2hBG (mmo/L)	17.30 (14.12, 20.90)	16.80 (13.33,20.28)	17.10 (14.10,20.25)	18.00 (15.03,21.58)	**0.003**
2hINS (mU/L)	25.88 (16.55, 41.21)	23.95 (15.52,38.61)	26.36 (17.30,43.69)	27.23 (16.44,41.06)	**0.036**
2hCP (ng/mL)	3.85 (2.83, 5.56)	3.75 (2.62,5.28)	3.91 (2.90,5.87)	3.87 (2.88,5.59)	0.080
Liver function tests					
AST (U/L)	19.00 (16.00, 25.00)	20.00 (17.00,25.00)	18.00 (15.00,24.00)	20.00 (16.00,26.00)	**0.037**
ALT (U/L)	21.00 (15.00, 30.00)	20.00 (14.00,29.00)	21.00 (15.25,29.00)	23.00 (17.00,32.00)	**0.002**
TB (umol/L)	14.50 (11.40, 18.40)	14.35 (11.43,18.48)	15.00 (12.22,18.45)	13.90 (10.80,18.38)	0.053
DB (umol/L)	2.80 (2.10, 3.50)	2.95 (2.30,3.70)	2.90 (2.30,3.50)	2.40 (1.80,3.10)	**<0.001**
IB (umol/L)	11.80 (9.12, 14.97)	11.50 (9.20,14.40)	12.10 (9.80,15.07)	11.50 (8.72,15.10)	0.125
TP (g/L)	69.10 (64.60, 72.88)	69.05 (64.80,72.40)	68.90 (63.85,72.70)	69.30 (65.20,73.38)	0.144
ALB (g/L)	43.40 (41.30, 45.40)	43.60 (41.42,45.30)	42.95 (41.10,45.40)	43.40 (41.40,45.40)	0.337
GLB (g/L)	25.50 (22.70, 28.50)	25.10 (22.70,28.20)	25.50 (22.60,28.40)	25.95 (23.30,28.98)	0.145
ALP (U/L)	86.00 (70.00, 104.00)	86.00 (69.00,104.00)	84.00 (69.00,100.00)	88.00 (72.00,107.00)	0.059
GGT (U/L)	24.95 (18.30, 37.53)	20.85 (15.62,29.90)	24.65 (17.90,34.70)	30.45 (22.83,47.32)	**<0.001**
CHE (ku/L)	8.32 (7.40, 9.30)	7.90 (7.11,8.67)	8.27 (7.38,9.15)	8.93 (8.03,9.91)	**<0.001**
TBA (μmol/L)	4.10 (2.60, 6.20)	4.00 (2.50,6.00)	3.95 (2.50,5.90)	4.40 (2.90,6.60)	0.055
LDH (U/L)	158.00 (138.00, 178.00)	164.00 (146.00,183.75)	156.00 (136.00,176.75)	154.00 (134.25,173.00)	**<0.001**
Renal function tests					
BUN (mmol/L)	5.43 (4.58, 6.35)	5.52 (4.67,6.39)	5.42 (4.46,6.36)	5.31 (4.58,6.32)	0.572
SCr (umol/L)	65.00 (56.00, 74.00)	64.00 (56.00,72.00)	66.00 (57.00,74.00)	66.00 (58.00,75.00)	**0.024**
eGFR [ml/(min·1.73m^2^)]	99.70 (93.05, 106.89)	97.87 (91.92,104.05)	99.27 (92.64,105.65)	102.76 (95.28,109.87)	**<0.001**
UA (umol/L)	321.00 (272.00, 371.00)	300.50 (254.25,349.00)	325.00 (274.00,368.75)	345.00 (291.25,396.75)	**<0.001**
Serum electrolytes					
Potassium (mmo/L)	3.94 (3.72, 4.15)	3.97 (3.79,4.18)	3.94 (3.71,4.16)	3.90 (3.68,4.09)	**0.004**
Sodium (mmo/L)	140.00 (138.00, 141.00)	140.00 (138.93,141.00)	140.00 (139.00,141.00)	139.00 (138.00,140.00)	**<0.001**
Chloride (mmo/L)	105.00 (103.00, 107.00)	105.00 (104.00,107.00)	105.00 (104.00,107.00)	104.00 (103.00,106.00)	**<0.001**
Calcium (mmo/L)	2.26 (2.18, 2.34)	2.26 (2.18,2.34)	2.25 (2.18,2.32)	2.26 (2.19,2.36)	0.190
Phosphate (mmo/L)	1.18 (1.06, 1.29)	1.16 (1.06,1.28)	1.17 (1.04,1.28)	1.20 (1.09,1.31)	**0.027**
Lipid profile					
TC (mmo/L)	4.41 (3.78, 5.09)	4.28 (3.62,4.96)	4.33 (3.78,4.94)	4.56 (3.94,5.23)	**<0.001**
TG (mmo/L)	1.50 (1.06, 2.18)	0.94 (0.78,1.10)	1.50 (1.29,1.73)	2.66 (2.10,3.92)	**<0.001**
HDL (mmo/L)	1.02 (0.87, 1.19)	1.20 (1.06,1.40)	1.02 (0.90,1.13)	0.87 (0.79,0.99)	**<0.001**
LDL (mmo/L)	2.81 (2.33, 3.29)	2.62 (2.13,3.14)	2.84 (2.43,3.29)	2.91 (2.46,3.37)	**<0.001**
Hormones					
T3 (ng/ml)	0.98 (0.86, 1.11)	0.98 (0.86,1.12)	0.97 (0.85,1.10)	0.98 (0.87,1.12)	0.645
T4 (ug/dL)	7.37 (6.30, 8.59)	7.50 (6.41,8.96)	7.23 (6.22,8.54)	7.29 (6.25,8.33)	0.080
TSH (uIU/mL)	2.36 (1.48, 3.61)	2.48 (1.44,3.83)	2.26 (1.48,3.46)	2.37 (1.49,3.60)	0.478
25(OH)D (nmol L)	15.14 (11.00, 19.54)	16.25 (11.38,20.04)	14.62 (11.01,18.99)	15.01 (10.79,19.70)	0.086
Complete blood count					
WBC (10^9/L)	5.76 (4.96, 6.84)	5.49 (4.73,6.50)	5.77 (4.99,6.77)	6.04 (5.14,7.14)	**<0.001**
RBC (10^12/L)	4.87 (4.58, 5.19)	4.77 (4.53,5.11)	4.92 (4.58,5.23)	4.95 (4.71,5.24)	**<0.001**
Hb (g/L)	150.00 (139.00, 159.00)	147.00 (137.00,156.00)	151.00 (139.00,160.00)	153.00 (143.07,161.00)	**<0.001**
Plt (10^9/L)	177.00 (147.00, 221.00)	177.50 (147.00,222.75)	172.50 (144.00,217.00)	182.00 (150.25,223.75)	0.409
LYMPH (10^9/L)	1.92 (1.57, 2.40)	1.89 (1.51,2.30)	1.90 (1.50,2.35)	2.05 (1.63,2.50)	**<0.001**
MONO (10^9/L)	0.37 (0.30, 0.45)	0.36 (0.30,0.45)	0.36 (0.30,0.44)	0.39 (0.30,0.47)	0.752
NEUT (10^9/L)	3.25 (2.65, 4.00)	3.09 (2.40,3.80)	3.31 (2.79,4.09)	3.40 (2.70,4.10)	**<0.001**
EO (10^9/L)	0.10 (0.09, 0.20)	0.10 (0.08,0.19)	0.10 (0.10,0.20)	0.10 (0.10,0.20)	**0.007**
BASO (10^9/L)	0.01 (0.00, 0.02)	0.01 (0.00,0.02)	0.01 (0.00,0.02)	0.01 (0.00,0.02)	0.949
Early-stage DKD, n (%)	404 (40.16)	108 (32.34)	123 (36.39)	173 (51.80)	**<0.001**

HTN, hypertension; MASLD, metabolic dysfunction-associated steatotic liver disease; CVD, cardiovascular disease; DR, diabetic retinopathy; DSPN, diabetic sensorimotor polyneuropathy; DPVD, diabetic peripheral vascular disease; FM, family history; DM, diabetes mellitus; NIAD, non-insulin antidiabetic drugs; LLA, lipid-lowering agents; Anti-inflamm, anti-inflammatory agents; Renoprot, renoprotective agents; AH, antihypertensive agents; SBP, systolic blood pressure; DBP, diastolic blood pressure; BMI, body mass index; HbA1c, glycated hemoglobin; FBG, fasting blood glucose; FINS, fasting insulin; FCP, fasting C-peptide; 2hBG, 2-h postprandial blood glucose; 2hINS, 2-h postprandial insulin; 2hCP, 2-h postprandial C-peptide; AST, aspartate aminotransferase; ALT, alanine aminotransferase; TB, total bilirubin; DB, direct bilirubin; IB, indirect bilirubin; TP, total protein; ALB, albumin; GLB, globulin; ALP, alkaline phosphatase; GGT, gamma-glutamyl transferase; CHE, cholinesterase; TBA, total bile acids; LDH, lactate dehydrogenase; BUN, blood urea nitrogen; SCr, serum creatinine; eGFR, estimated glomerular filtration rate; UA, uric acid; TC, total cholesterol; TG, triglycerides; HDL, high-density lipoprotein; LDL, low-density lipoprotein; T3, triiodothyronine; T4, thyroxine; TSH, thyroid-stimulating hormone; 25(OH)D, 25-hydroxyvitamin D; WBC, white blood cell; RBC, red blood cell; Hb, hemoglobin; Plt, platelet; LYMPH, lymphocyte; MONO, monocyte; NEUT, neutrophil; EO, eosinophil; BASO, basophil; T1, tertile 1(AIP<0.053); T2, tertile 2 (0.053 ≤ AIP<0.295); T3, tertile 3 (AIP≥0.295). NIAD: including biguanides, sulfonylureas, glinides, α-glucosidase inhibitors, DPP-4 inhibitors, SGLT2 inhibitors, TZDs, and GLP-1 receptor agonists; LLA: including statins and fibrates; Anti-inflamm: including nonsteroidal anti-inflammatory drugs (NSAIDs); Renoprot: including finerenone, Huangkui capsule, and JinShuiBao; AH: including ACEIs, ARBs, CCBs, β-blockers, and diuretics. Bold values indicate *P* < 0.05, which was considered statistically significant.

### Exposure variable and outcome definition

The exposure variable in this study was AIP, calculated using the following formula: AIP = log [TG (mmol/L) / HDL-C (mmol/L)] (28). The study outcome was the occurrence of ES-DKD during follow-up. According to the KDIGO Clinical Practice Guideline for Chronic Kidney Disease (29) and the World Health Organization guideline for diabetes (26), ES-DKD (stages 1–2) was defined in this study as the first occurrence of eGFR ≥ 60 mL/(min·1.73 m^2^) with a UACR ≥ 30 mg/g, confirmed by subsequent follow-up assessments. Non-DKD was defined as patients who maintained UACR < 30 mg/g and eGFR ≥ 60 mL/ (min·1.73 m^2^) throughout the follow-up period, representing the diabetic population without significant kidney injury.

### Sample size estimation

The sample size was determined based on the events per variable (EPV) criterion. For an anticipated 5–10 predictor variables, at least 50–100 ES-DKD events were required (30). According to literature reports (31), the incidence of ES-DKD in patients with T2DM is approximately 20–40%, yielding a calculated requirement of 125–500 patients. Considering a potential 20% loss of data, the target for the training cohort was adjusted to 150–600 patients. Accordingly, based on a 7:3 split ratio, the validation cohort was planned to include at least 64–214 patients for validation. A total of 1,006 patients with T2DM (704 in the training cohort and 302 in the validation cohort) were ultimately enrolled in this study, exceeding the calculated required sample size.

### Definitions of complications and comorbidities

The definitions of complications and comorbidities in this study were primarily based on the International Classification of Diseases, Tenth Revision codes. Specifically, hypertension (HTN) was identified by self-report or codes I10-I15; MASLD was defined as code K76.0 or based on the physician’s original diagnostic text. Cardiovascular disease (CVD) was defined to encompass codes I20-I25 (ischemic heart disease) and I60-I69 (cerebrovascular disease), or based on patient self-report. In addition, diabetic retinopathy (DR), diabetic sensorimotor polyneuropathy (DSPN), and diabetic peripheral vascular disease (DPVD) were defined as codes E11.3, E11.42, and E11.5, respectively.

### Statistical analysis

A total of 1,006 patients were divided into three groups based on the tertiles of baseline AIP. In addition, patients were divided into two groups according to whether ES-DKD occurred during follow-up. For baseline characteristics across these groups, continuous variables with a normal distribution were presented as mean ± standard deviation (Mean ± SD), and comparisons between groups were performed using analysis of variance (ANOVA) or Student’s t-test. Continuous variables with a non-normal distribution were presented as median with interquartile range [M (P25-P75)], and comparisons between groups were performed using the Kruskal-Wallis test or Mann–Whitney *U* test. Categorical variables were presented as numbers and percentages, and comparisons between groups were performed using the chi-square test or Fisher’s exact test. Using the lowest AIP tertile group as the reference, Cox proportional hazards models were employed to assess the association between AIP tertiles and the outcome event, with hazard ratios (HRs) and their 95% confidence intervals (CIs) calculated. Model I was an unadjusted model. Model II was adjusted for the following potential confounders (selected based on significant differences between patients with non-DKD and ES-DKD): gender, career, smoking, family history of diabetes mellitus (FM of DM), insulins, anti-inflammatory drugs (Anti-inflamm), renoprotective agents (Renoprot), antihypertensive drugs (AH), systolic blood pressure (SBP), diastolic blood pressure (DBP), body mass index (BMI), glycated hemoglobin (HbA1c), fasting blood glucose (FBG), fasting C-peptide (FCP), 2-h postprandial blood glucose (2hBG), 2-h postprandial C-peptide (2hCP), aspartate aminotransferase (AST), alanine aminotransferase (ALT), total protein (TP), globulin (GLB), alkaline phosphatase (ALP), gamma-glutamyl transferase (GGT), total bile acids (TBA), lactate dehydrogenase (LDH), uric acid (UA), sodium, chloride, triglycerides (TG), high-density lipoprotein cholesterol (HDL), white blood cell count (WBC), red blood cell count (RBC), hemoglobin (Hb), neutrophils (NEUT), and eosinophils (EO). Furthermore, subgroup analyses and likelihood ratio tests for interaction were performed to evaluate the robustness of the findings.

### Restricted cubic spline (RCS) analysis and survival analysis

To explore the potential dose–response relationship between AIP levels and the outcome variable, a RCS regression model was constructed, with the number of knots determined by minimizing the Bayesian information criterion. A likelihood ratio test was used to assess the statistical significance of nonlinear trends (32). In addition, survival curves stratified by AIP tertiles were plotted using the Kaplan–Meier (KM) method, and comparisons between groups were performed using the log-rank test (33).

### Boruta algorithm

The Boruta algorithm was employed to evaluate the importance of AIP as a predictor variable. This algorithm creates randomly shuffled shadow features as noise benchmarks and uses the Z-scores derived from a random forest model to assess the importance of each real feature. During iterative comparisons, if the Z-score of a real feature was significantly higher than the maximum Z-score among all shadow features, it was classified as an “important feature” in the green region and retained in the model. If it was significantly lower than this threshold, it was classified as an “unimportant feature” in the red region and excluded. If the difference did not reach statistical significance, the feature was labeled as a “tentative feature” in the yellow region. Through this process, the final set of variables that significantly contributed to the model was identified (34).

### Construction and validation of the prediction model

To robustly identify key predictors beyond traditional analysis, SML methods were introduced. The dataset was randomly divided into a training cohort (*n* = 704) and a validation cohort (*n* = 302) at a 7:3 ratio for constructing and validating the prognostic prediction models, ensuring balanced baseline characteristics between the two groups (see Supplementary Table S4). First, in the training cohort, variables with significant differences in univariate analysis (*p* < 0.05) were included in LASSO regression to select variables associated with ES-DKD risk. Subsequently, backward stepwise selection (retention threshold: *p* < 0.05) was applied to identify the final set of predictors. Variance inflation factors (VIF) were calculated, and variables with VIF > 5 were excluded to avoid multicollinearity.

To visualize the clustering distribution of the aforementioned independent risk factors between the ES-DKD and non-DKD cohorts, unsupervised hierarchical clustering was performed using Euclidean distance combined with Ward’s linkage method. All continuous variables were standardized using Z-score normalization to eliminate scale differences. The heatmap was generated using the “ComplexHeatmap” package in R software, with dendrograms illustrating the clustering structure and a color gradient representing the levels of standardized risk factors (red indicating high values, blue indicating low values).

The selected variables were incorporated into six prognostic SML algorithms for survival analysis, including random survival forest (RSF), lasso-penalized Cox proportional hazards model (Lasso_Cox), CoxBoost, extreme gradient boosting (XGBoost), supervised principal components (superpc), and partial least squares regression for the Cox model (plsRcox), to assess the risk of ES-DKD in patients with T2DM. A detailed description of the SML algorithms, hyperparameter tuning, and validation procedures is provided in ESM2. Time-dependent area under the curve (AUC) was used to evaluate the predictive performance (dynamic discriminative ability) of each prognostic SML model at different time points. Receiver operating characteristic (ROC) curves and their AUC values were employed to assess the static discriminative ability of each model. Calibration curves were used to examine the accuracy of the predicted absolute risks (calibration). Decision curve analysis (DCA) was applied to evaluate the clinical net benefit of the models. Through the above process, the model with the best performance was selected.

### Web-based application tool

To provide a more intuitive reference for clinical practice, this study developed an online early warning system based on the multivariate Cox proportional hazards regression model to facilitate the prediction of future ES-DKD risk. This tool features dynamic monitoring capabilities and offers evidence-based clinical decision support for patients with ES-DKD.

A *p*-value < 0.05 was considered statistically significant. Statistical analyses and visualizations were performed using R (version 4.3.1), Zstats^1^, and PCPM (V3.22, Jingding Medical Technology Co., Ltd.).

## Results

### Baseline characteristics of the study population

This study ultimately included data from 1,006 patients with T2DM. Among these patients, 64.71% were male, and the mean age was 56.53 years. Patients were divided into three groups according to the tertiles of baseline AIP at admission: tertile 1 group (AIP < 0.053, *n* = 334), tertile 2 group (0.053 ≤ AIP < 0.295, *n* = 338), and tertile 3 group (AIP ≥ 0.295, *n* = 334). As the AIP tertile level increased, the prevalence of early-stage DKD progressively increased (32.34% vs. 36.39% vs. 51.80%, *p* < 0.001). Compared with the other groups, patients in the tertile 3 group were younger, had a shorter disease duration, a higher proportion of male sex, and a higher proportion of MASLD, whereas the proportions of HTN, CVD, and DR were lower (*p* < 0.05). In addition, patients in the tertile 3 group had higher levels of lipid-lowering agent (LLA) use, DBP, BMI, HbA1c, FBG, FINS, FCP, 2hBG, 2hINS, ALT, AST, GGT, CHE, SCr, eGFR, UA, phosphate, TC, TG, LDL, WBC, RBC, Hb, LYMPH, NEUT, and EO (*p* < 0.05), while levels of antihypertensive drug (AH) use, DB, LDH, potassium, sodium, chloride, and HDL were lower (*p* < 0.01) (see Table 1).

During a median follow-up period of 49 months, a total of 404 cases of ES-DKD were documented. Significant differences were observed between patients with non-DKD and those with early-stage DKD in terms of sex, career, smoking, FM of DM, medication use (insulins, anti-inflamm, renoprot, and AH), blood pressure, BMI, glucose metabolism indicators (HbA1c, FBG, FCP, 2hBG, and 2hCP), liver function parameters (ALT, AST, TP, GLB, ALP, GGT, TBA, LDH), renal function parameters (UA), serum electrolytes (sodium and chloride), lipid profiles (TG and HDL), routine blood parameters (WBC, RBC, Hb, NEUT, and EO), and AIP (*p* < 0.05). Additional baseline characteristics of the two groups are detailed in Supplementary Table S2.

### Cox proportional hazards regression and the subgroup analysis

Unadjusted and adjusted Cox proportional hazards regression analyses (adjusted for significant differences between patients with non-DKD and ES-DKD, as shown in Supplementary Table S2) were performed to evaluate the association between AIP and the occurrence of ES-DKD. The results showed that when AIP was analyzed as a continuous variable, the adjusted HR was 1.74 (95% CI: 1.19–2.54, *p* = 0.004). Using the AIP tertile 1 group as the reference, the adjusted HR for the AIP tertile 3 group was 1.33 (95% CI: 1.01–1.74, *p* = 0.039), and the test for trend was statistically significant (*P* for trend < 0.05). These findings confirm that AIP is independently associated with the occurrence of ES-DKD (Table 2).

**Table 2:** Multivariable cox regression analyses for early-stage diabetic kidney disease.

AIP	Case/total	Unadjusted model I		Adjusted model II	
		HR (95% CI)	*P*	HR (95% CI)	*P*
Per 1 unit increase	404/1006	2.12 (1.58–2.84)	**<0.001**	1.74 (1.19–2.54)	**0.004**
Tertiles					
T1	108/334	1.00 (Reference)		1.00 (Reference)	
T2	123/338	1.10 (0.85–1.42)	0.474	0.98 (0.75–1.29)	0.896
T3	173/334	1.57 (1.23–2.00)	**<0.001**	1.33 (1.01–1.74)	**0.039**
*P* for trend		2.35 (1.52–3.63)	**<0.001**	1.75 (1.07–2.86)	**0.027**

AIP, atherogenic index of plasma; HR, hazard ratio; CI, confidence interval; T1, tertile 1(AIP<0.053); T2, tertile 2 (0.053 ≤ AIP<0.295); T3, tertile 3 (AIP≥0.295).

**Figure 2:**
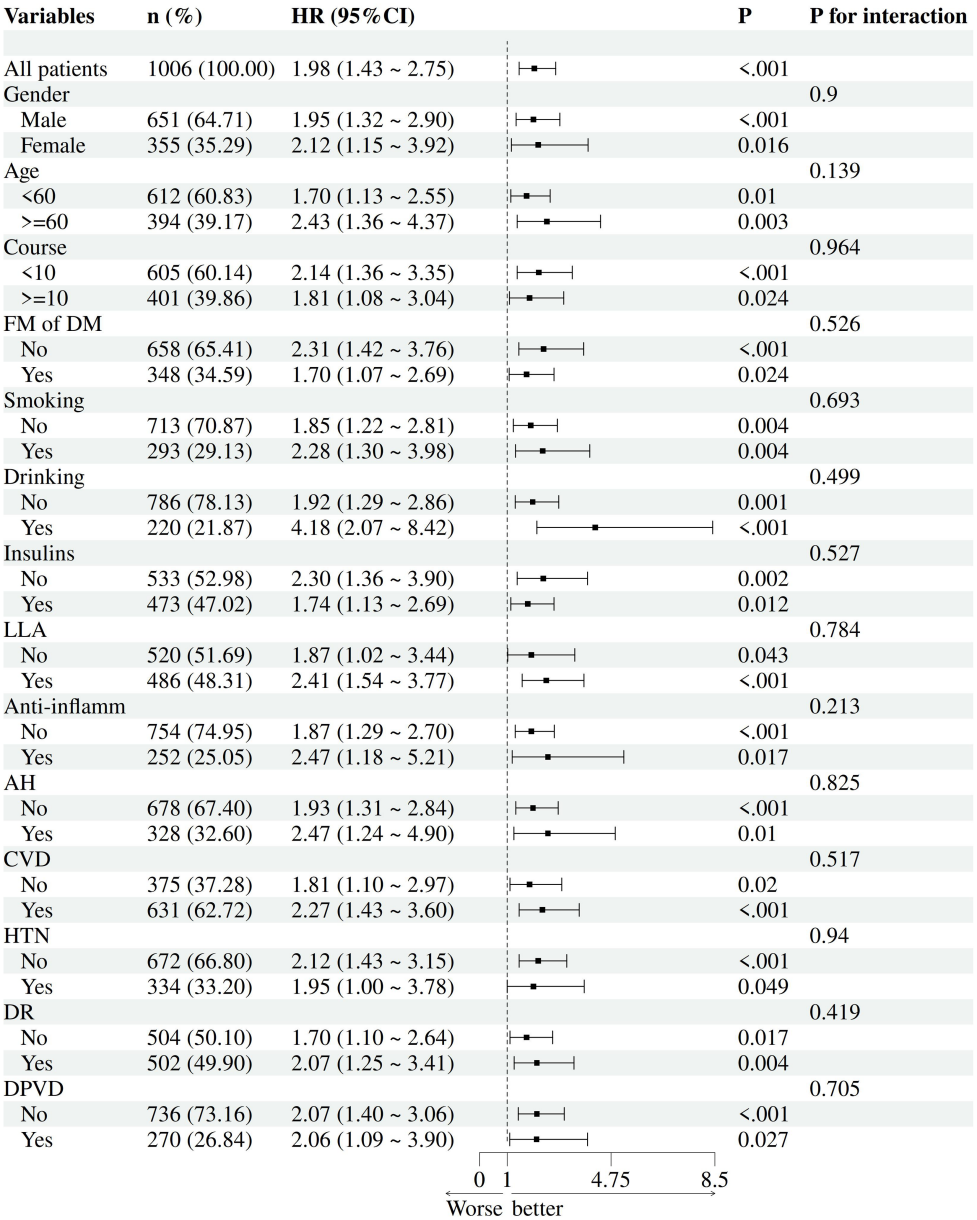
Cox proportional hazards regression and subgroup analysis forest plot for the association of atherogenic index of plasma (AIP) with early-stage diabetic kidney disease (ES-DKD) risk (adjusted). HR, hazard ratio; CI, confidence interval; FM of DM, family history of diabetes mellitus; LLA, lipid-lowering agents; Anti-inflamm, anti-inflammatory agents; AH, antihypertensive agents; CVD, cardiovascular disease; HTN, hypertension; DR, diabetic retinopathy; DPVD, diabetic peripheral vascular disease.

Subgroup analyses were performed based on potential influencing factors, including demographic characteristics (age and sex), disease duration, lifestyle factors and family history (FM of DM, smoking, and drinking), medication use (insulins, LLA, anti-inflamm, and AH), and comorbidities (CVD, HTN, DR, and DPVD), to investigate whether AIP has prognostic value for ES-DKD across different patient subgroups. The results showed that elevated AIP levels were significantly associated with an increased risk of ES-DKD across all subgroups (unadjusted and adjusted HR > 1, *p* < 0.05, *P* for interaction > 0.05; Supplementary Figure S1 and Figure 2). Supplementary Table S3 presents the associations between AIP and the risk of ES-DKD in other subgroup influencing factors, including ethnicity, occupation, FM of HTN, non-insulin antidiabetic drugs (NIAD), HbA1c, DSPN, and MASLD. Collectively, these findings suggest that AIP is an effective prognostic indicator for predicting the occurrence of ES-DKD, supporting the robustness of this association.

### RCS analysis and survival analysis of the association between AIP levels and ES-DKD

**Figure 3:**
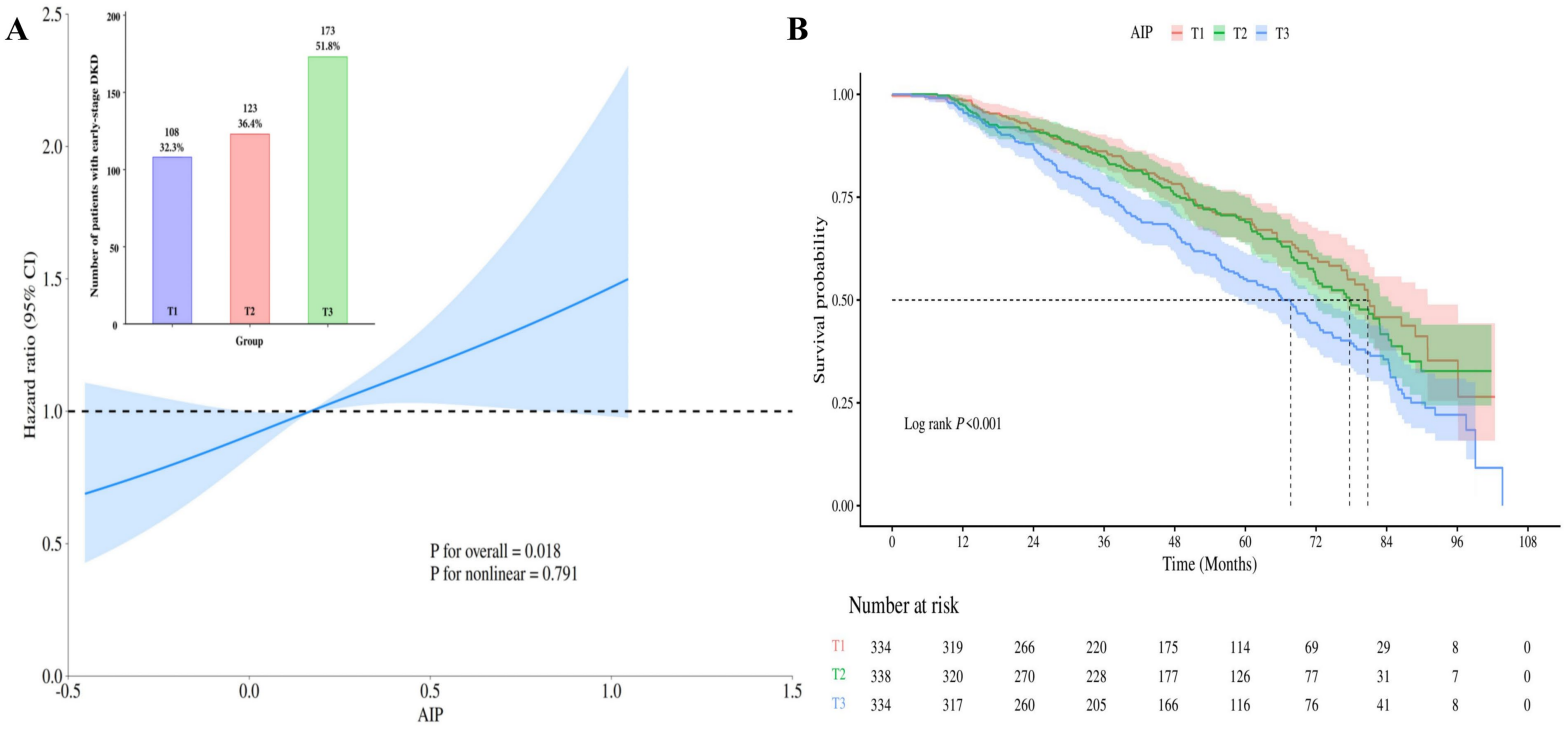
Restricted cubic spline analyses and Kaplan–Meier curves for the association between atherogenic index of plasma (AIP) and early-stage diabetic kidney disease (ES-DKD). **(A)** Restricted cubic spline analyses (adjusted); **(B)** Kaplan–Meier curves. T1, tertile 1(AIP < 0.053); T2, tertile 2 (0.053 ≤ AIP < 0.295); T3, tertile 3 (AIP ≥ 0.295).

To further explore the potential nonlinear relationship between AIP as a continuous variable and ES-DKD, RCS analysis was performed. The results demonstrated a linear positive association between elevated AIP levels and the risk of the outcome (*P* for overall < 0.05 and *P* for nonlinear > 0.05). After both unadjusted and adjusted analyses for potential risk factors (all variables with significant differences in Supplementary Table S2), AIP remained significantly associated with ES-DKD (Supplementary Figure S2 and Figure 3A). Kaplan–Meier survival analysis revealed significant differences in the risk of ES-DKD among the three groups stratified by AIP tertiles (log-rank *p* < 0.001), with AIP levels positively correlated with the risk of ES-DKD (Figure 3B).

### Importance ranking of factors associated with ES-DKD using the Boruta algorithm

**Figure 4:**
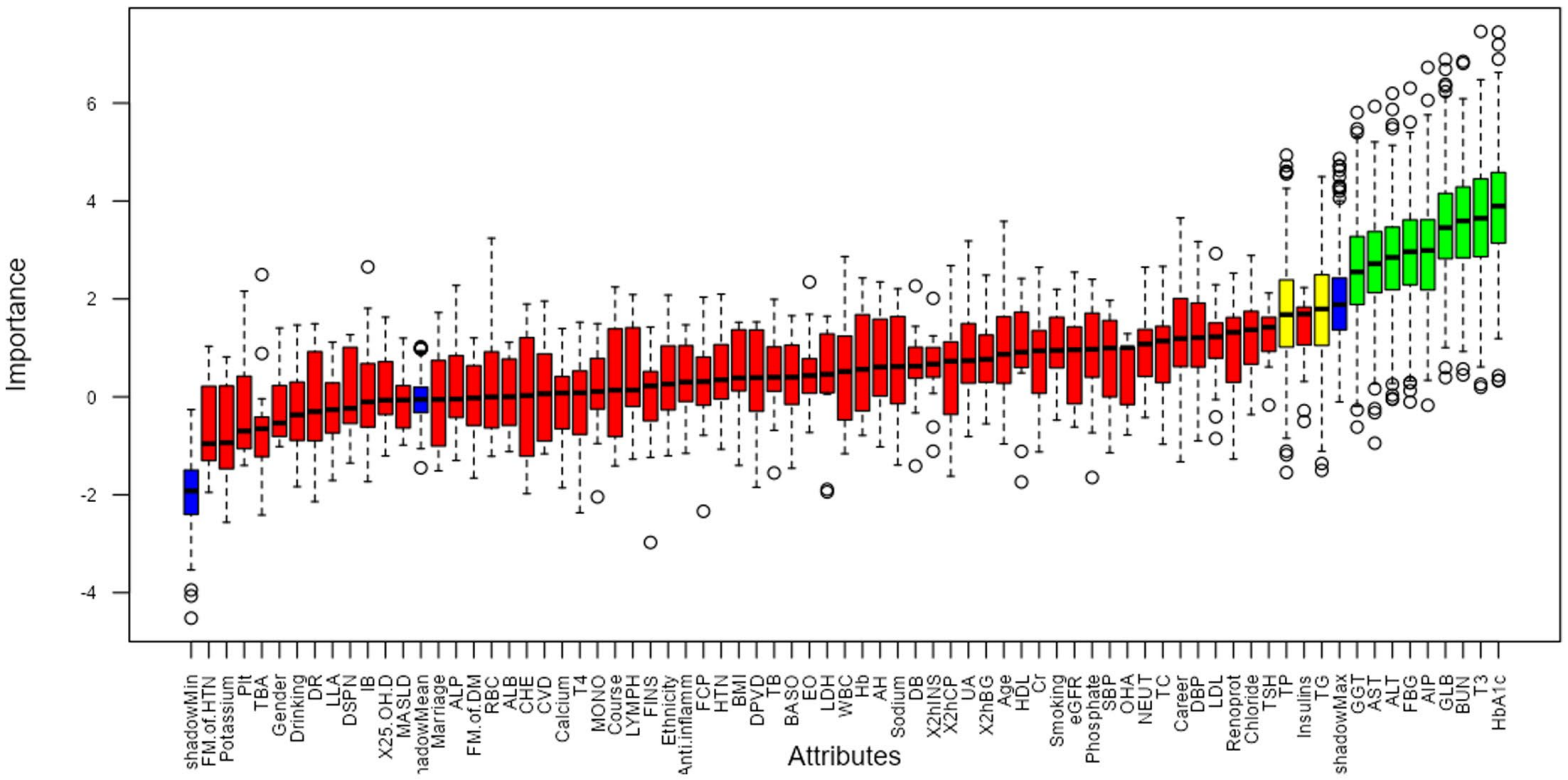
Importance ranking of potential risk factors for early-stage diabetic kidney disease (ES-DKD) by Boruta algorithm. The horizontal axis is the name of each variable, and the vertical axis is the *Z* value of each variable. The box plot shows the *Z* value of the importance score for each variable during model calculation. Among them, green boxes represent important variables, red boxes represent unimportant variables, and yellow boxes represent potentially important variables. HTN, hypertension; MASLD, metabolic dysfunction-associated steatotic liver disease; CVD, cardiovascular disease; DR, diabetic retinopathy; DSPN, diabetic sensorimotor polyneuropathy; DPVD, diabetic peripheral vascular disease; FM, family history; DM, diabetes mellitus; NIAD, non-insulin antidiabetic drugs; LLA, lipid-lowering agents; Anti-inflamm, anti-inflammatory agents; Renoprot, renoprotective agents; AH, antihypertensive agents; SBP, systolic blood pressure; DBP, diastolic blood pressure; BMI, body mass index; HbA1c, glycated hemoglobin; FBG, fasting blood glucose; FINS, fasting insulin; FCP, fasting C-peptide; 2hBG, 2-h postprandial blood glucose; 2hINS, 2-h postprandial insulin; 2hCP, 2-h postprandial C-peptide; AST, aspartate aminotransferase; ALT, alanine aminotransferase; TB, total bilirubin; DB, direct bilirubin; IB, indirect bilirubin; TP, total protein; ALB, albumin; GLB, globulin; ALP, alkaline phosphatase; GGT, gamma-glutamyl transferase; CHE, cholinesterase; TBA, total bile acids; LDH, lactate dehydrogenase; BUN, blood urea nitrogen; SCr, serum creatinine; eGFR, estimated glomerular filtration rate; UA, uric acid; TC, total cholesterol; TG, triglycerides; HDL, high-density lipoprotein; LDL, low-density lipoprotein; T3, triiodothyronine; T4, thyroxine; TSH, thyroid-stimulating hormone; 25(OH)D, 25-hydroxyvitamin D; WBC, white blood cell; RBC, red blood cell; Hb, hemoglobin; Plt, platelet; LYMPH, lymphocyte; MONO, monocyte; NEUT, neutrophil; EO, eosinophil; BASO, basophil.

In the feature selection results of the Boruta algorithm, variables in the green region were identified as important factors that play a significant role in the prediction model. Variables in the yellow region were considered tentative factors that may be associated with the occurrence of the clinical outcome. Variables in the red region were identified as unimportant factors. The results indicated that among the 72 variables, AIP ranked fifth in the green region and demonstrated a high importance score (*Z*-score), confirming it as one of the key factors for predicting the occurrence of ES-DKD in patients with T2DM (Figure 4).

### Construction and validation of SML prediction models incorporating AIP

Given that the above findings indicate AIP is an important predictor of ES-DKD in patients with T2DM, this section further constructs SML prediction models incorporating AIP, aiming to translate it into a clinically applicable prognostic tool to facilitate risk assessment and early intervention.

#### Feature selection and validation

**Figure 5:**
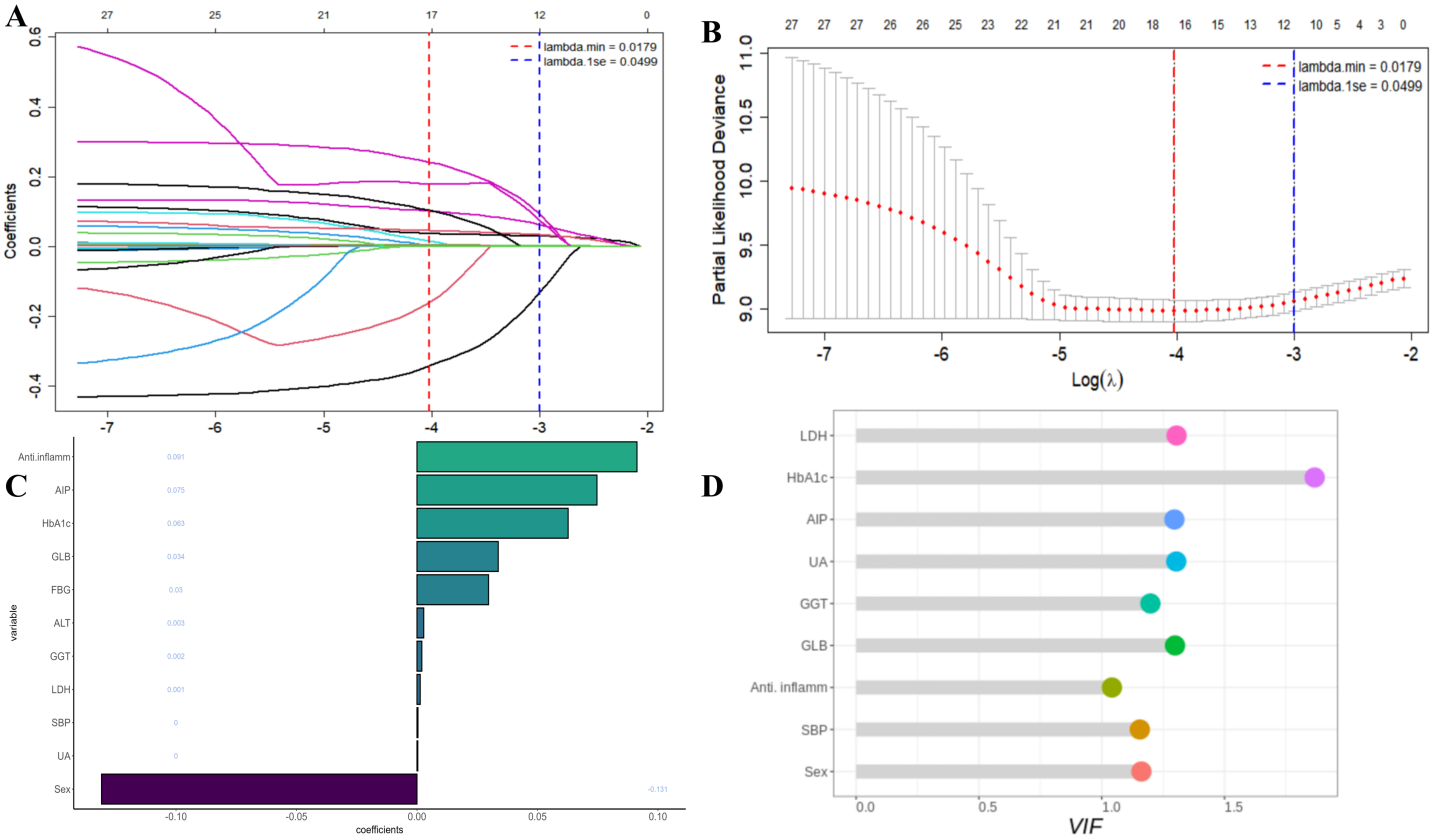
Feature selection for predicting early-stage diabetic kidney disease (ES-DKD) in patients with type 2 diabetes mellitus. **(A)** LASSO coefficient profiles of the candidate features. Each curve represents the trajectory of a coefficient as the regularization parameter *λ* changes. **(B)** 10-fold cross-validation for tuning parameter selection in LASSO regression. The partial likelihood deviance is plotted against log(λ). The left dashed vertical line indicates the λ value that yields the minimum deviance (λ_min = 0.0179), while the right dashed vertical line indicates the λ value within one standard error of the minimum (λ_1se = 0.0499). **(C).** Coefficient values of the 11 features selected at λ_1se. **(D)** Variance inflation factor (VIF) analysis of the final selected predictors, confirming the absence of multicollinearity (VIF < 5). LASSO, least absolute shrinkage and selection operator; VIF, variance inflation factor.

**Figure 6:**
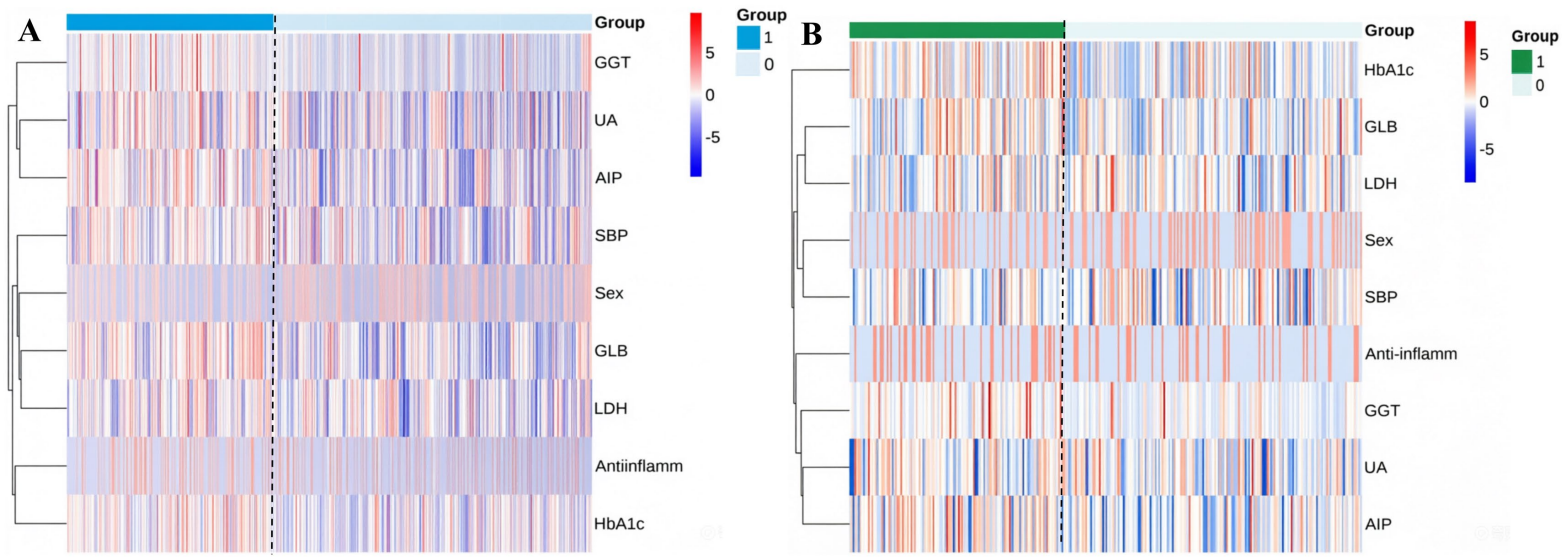
Clustering heatmap of the selected predictors in the training **(A)** and validation cohorts **(B)**. The heatmap visualizes the distribution patterns of the nine independent predictors (Sex, SBP, Anti-inflamm, GLB, GGT, UA, AIP, HbA1c, and LDH) between the non-DKD (Group 0) and ES-DKD (Group 1) groups. Rows represent the individual patients, columns represent the selected variables. Red indicates higher levels of the variable, while blue indicates lower levels. The dendrograms on the top and left sides represent the hierarchical clustering results for variables and patients, respectively. ES-DKD, early-stage diabetic kidney disease; SBP, systolic blood pressure; GLB, globulin; GGT, gamma-glutamyl transferase; UA, uric acid; AIP, atherogenic index of plasma; HbA1c, glycated hemoglobin; LDH, lactate dehydrogenase; Anti-inflamm, anti-inflammatory agents.

A total of 1,006 patients were divided into a training cohort (*n* = 704) and a validation cohort (*n* = 302). No significant differences in clinical characteristics were observed between the two cohorts, indicating good comparability (Supplementary Table S4). In the training cohort, variables with significant differences in univariate analysis (Supplementary Table S5) were included in LASSO regression to select variables associated with the risk of ES-DKD, yielding 11 features (Figures 5A–C). Subsequently, backward stepwise selection was applied to ultimately identify nine predictors for training the SML model, namely sex, SBP, anti.inflamm, GLB, GGT, UA, AIP, HbA1c, and LDH. The VIFs for these variables were all less than 5, indicating the absence of multicollinearity (Supplementary Table S6, Supplementary Figure S3, and Figure 5D). Cluster heatmaps were used to display the distribution patterns of these nine independent factors between the non-DKD and ES-DKD groups in both the training and validation cohorts (Figure 6). These visualization results showed a denser clustering of risk factor values in the ES-DKD cohort.

#### Development and validation of SML models

In the context of the above multivariable analysis, SML methods were further employed to develop prognostic prediction models incorporating AIP in the training cohort. A total of six prognostic SML algorithms for survival analysis were applied, including RSF, Lasso_Cox, CoxBoost, XGBoost, superpc, and plsRcox. Among these, the variable importance analysis from the RSF algorithm confirmed that AIP was one of the important predictors, highlighting its value in prognostic assessment (Supplementary Figure S4).

**Figure 7:**
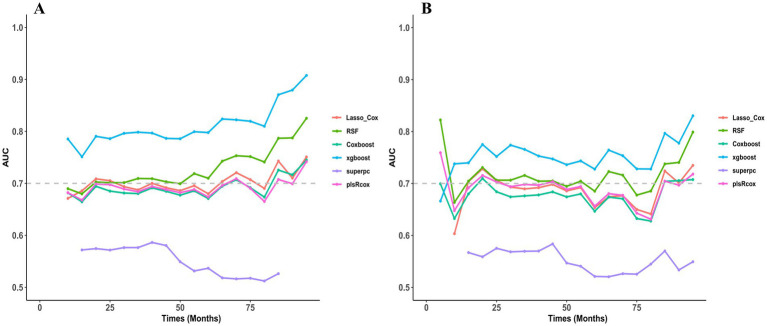
Time-dependent AUC curves of six prognostic machine learning models for predicting early-stage diabetic kidney disease (ES-DKD) in patients with type 2 diabetes mellitus. **(A)** Training cohort. **(B)** Validation cohort. The x-axis represents follow-up time (months), and the y-axis represents the time-dependent AUC value. The performance of six models (RSF, Lasso_Cox, coxboost, XGBoost, superpc, and plsRcox) is displayed across the follow-up period. Among all models, the XGBoost algorithm demonstrates the optimal predictive performance. AUC, area under the curve; RSF, random survival forest; Lasso_Cox, lasso-penalized Cox proportional hazards model; XGBoost, extreme gradient boosting; superpc, supervised principal components; plsRcox, partial least squares regression for Cox model.

**Figure 8:**
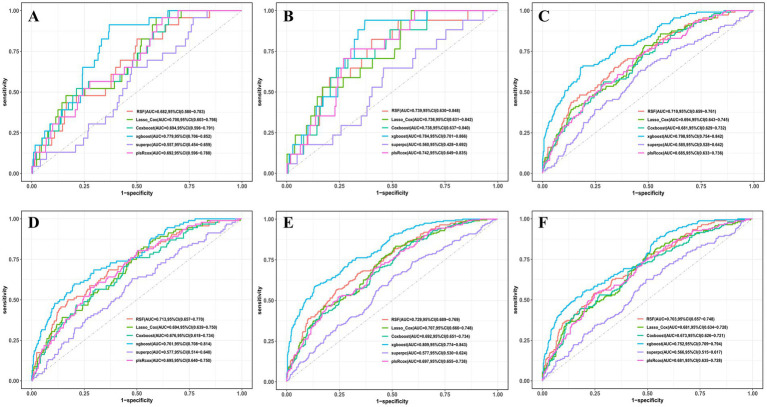
ROC curves of six machine learning models for predicting early-stage diabetic kidney disease (ES-DKD) at different time points in the training cohort and validation cohort. **(A,C,E)** respectively, display the ROC curves of six machine learning models for predicting ES-DKD at 1, 3, and 5 years of follow-up in the training cohort; **(B,D,F)** correspond to the results in the validation cohort. ES-DKD, early-stage diabetic kidney disease; RSF, random survival forest; Lasso_Cox, lasso-penalized Cox proportional hazards model; XGBoost, extreme gradient boosting; superpc, supervised principal components; plsRcox, partial least squares regression for Cox model.

**Figure 9:**
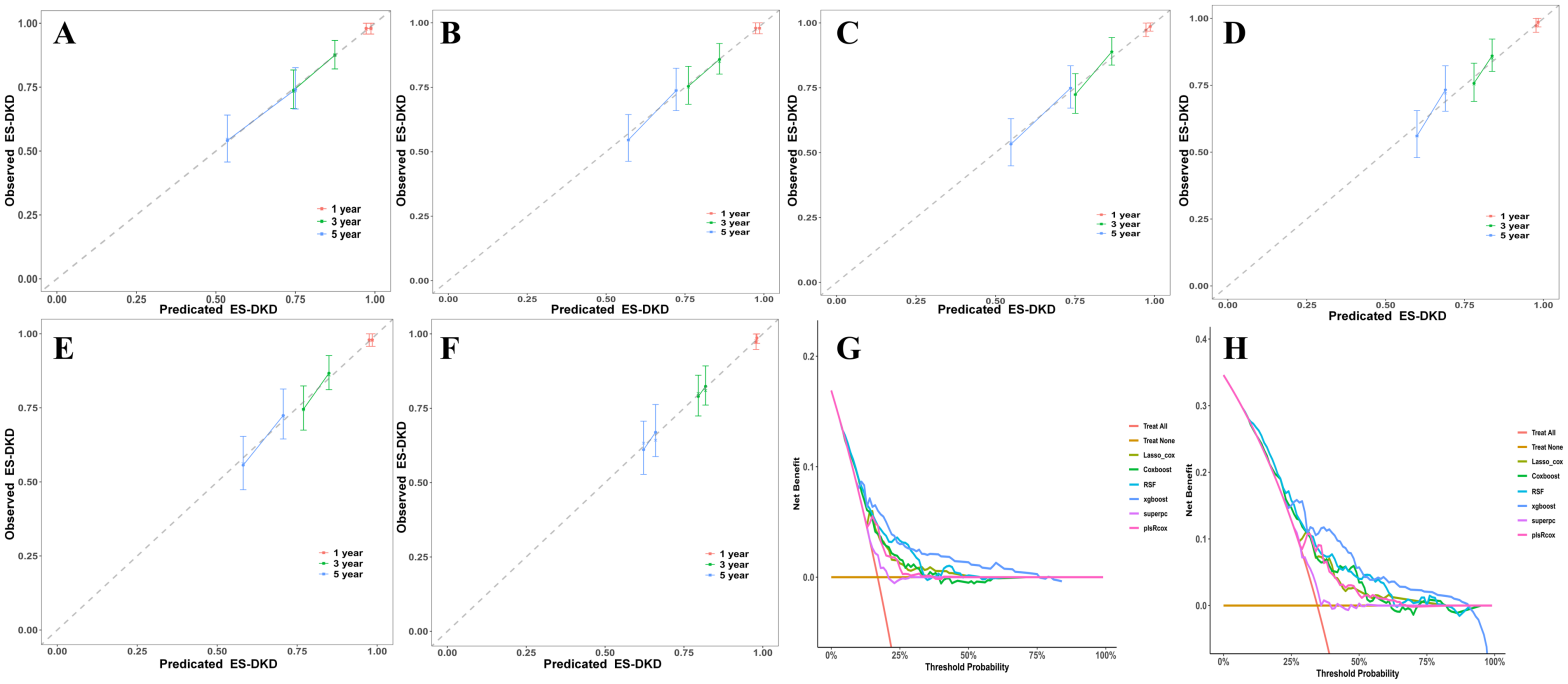
Calibration curves and decision curve analysis of six prognostic machine learning models for predicting early-stage diabetic kidney disease (ES-DKD) in the validation cohort. **(A–F)** Calibration curves for RSF, Lasso_Cox, coxboost, XGBoost, superpc, and plsRcox models at 1, 3, and 5 years of follow-up, respectively. The *x*-axis represents the predicted probability of ES-DKD, and the *y*-axis represents the observed probability. The diagonal dashed line indicates perfect calibration, where predicted probabilities equal observed probabilities. The solid curves represent the actual performance of the models, with closer proximity to the diagonal line indicating better calibration. **(G,H)** Decision curve analysis of the six machine learning models at 3 and 5 years of follow-up. The *x*-axis represents the threshold probability, and the *y*-axis represents the net benefit. The horizontal line represents the assumption that no patients have ES-DKD (intervention for none), and the diagonal line represents the assumption that all patients have ES-DKD (intervention for all). RSF, random survival forest; Lasso_Cox, lasso-penalized Cox proportional hazards model; XGBoost, extreme gradient boosting; superpc, supervised principal components; plsRcox, partial least squares regression for Cox model.

Figure 7 presents the time-dependent AUC curves of the six SML algorithms in both the training (Figure 7A) and validation (Figure 7B) cohorts. Among all models, the prognostic prediction model based on the XGBoost algorithm exhibited the best overall predictive performance. ROC curves were further plotted at three time points: 1, 3, and 5 years. The results showed that compared with other algorithmic models, the XGBoost-based prognostic prediction model achieved the highest AUC at all three time points, with values of 0.779, 0.798, and 0.809 in the training cohort (Figures 8A,C,E), and 0.784, 0.761, and 0.752 in the validation cohort (Figures 8B,D,F), respectively. Calibration curves demonstrated that the predicted probabilities for 1-year, 3-year, and 5-year ES-DKD risk from each SML model were in good agreement with the observed probabilities in both the training cohort (Supplementary Figures S5) and the validation cohort (Figures 9A–F). DCA indicated that the XGBoost-based prognostic prediction model provided the highest net benefit at the 3-year and 5-year time points in both the training cohort (Supplementary Figures S5) and the validation cohort (Figures 9G,H), demonstrating strong clinical utility.

#### Application of the prognostic prediction model

**Figure 10:**
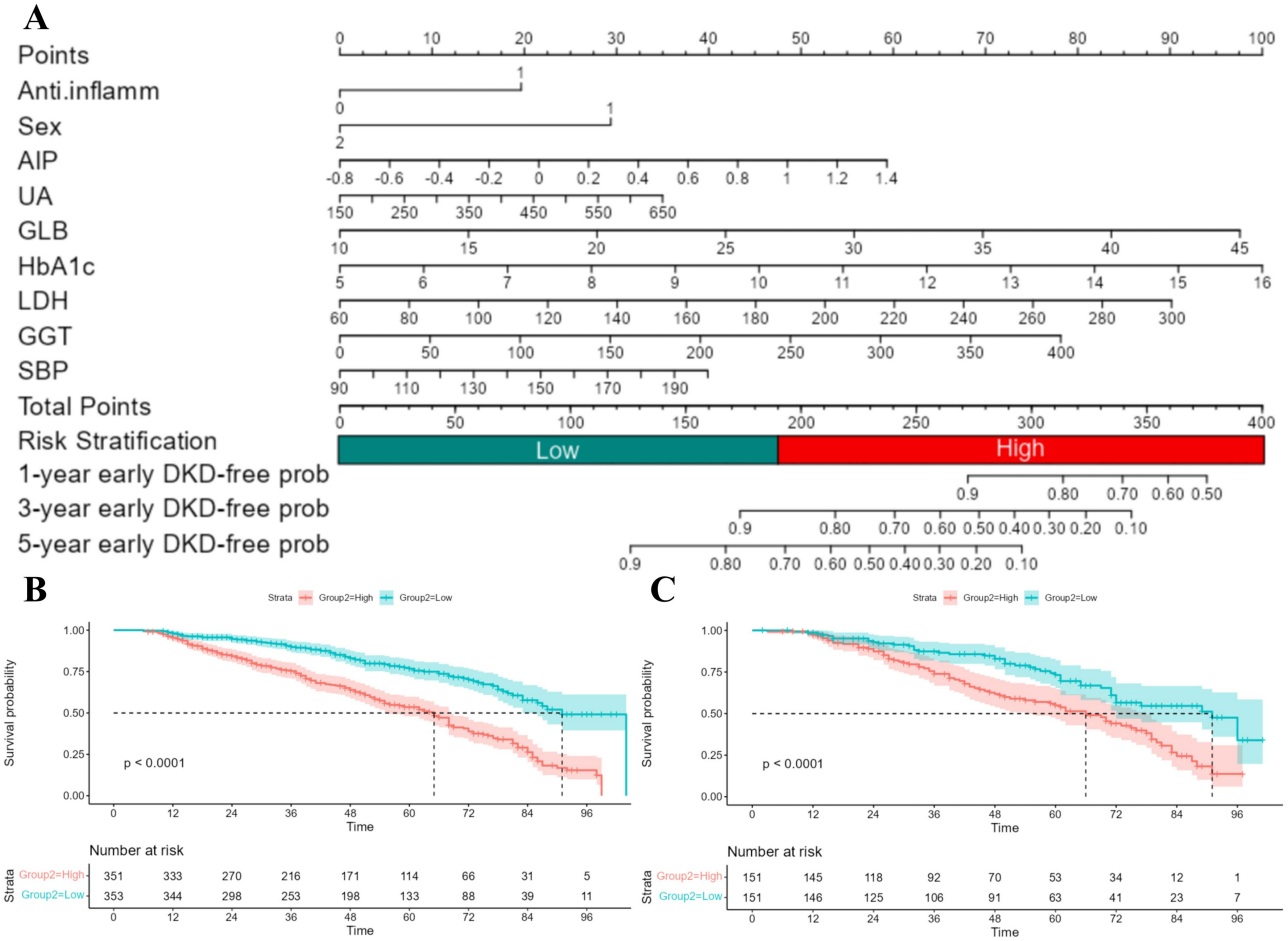
Nomogram and Kaplan–Meier survival curves for predicting early-stage diabetic kidney disease (ES-DKD) in patients with type 2 diabetes mellitus. **(A)** Nomogram for predicting the probability of ES-DKD at 1, 3, and 5 years. The nomogram was constructed based on the multivariate Cox proportional hazards regression model. It integrates nine independent predictors. Points are assigned to each predictor based on its value, and the total points correspond to the ES-DKD-free survival probability at each time point. **(B)** Kaplan–Meier survival curves for risk stratification in the training cohort. **(C)** Kaplan–Meier survival curves for the validation cohort. ES-DKD, early-stage diabetic kidney disease; SBP, systolic blood pressure; GLB, globulin; GGT, gamma-glutamyl transferase; UA, uric acid; AIP, atherogenic index of plasma; HbA1c, glycated hemoglobin; LDH, lactate dehydrogenase.

To provide a more intuitive reference for clinical practice, the prognostic prediction model, which was based on a multivariable Cox regression incorporating the nine independent predictors, was visualized as a nomogram (Figure 10A), and an online early warning system was developed to facilitate future risk prediction^2^. This tool supports dynamic monitoring and provides support for evidence-based clinical decision-making in patients with ES-DKD (Supplementary Figure S6). We calculated the AUCs for predicting 1-, 3-, and 5-year ES-DKD risk in both the training and validation cohorts. In the training cohort, the AUCs of the Cox model were 0.703, 0.719, and 0.704, respectively; in the validation cohort, they were 0.716, 0.708, and 0.706, respectively. Compared with the XGBoost model, this Cox-based tool shows a modest performance trade-off, but offers the advantages of stronger interpretability and greater ease of bedside application. In addition, to validate the prognostic value of the prognostic prediction model, Kaplan–Meier survival curve analysis was performed to assess the model’s ability to stratify patients with T2DM for ES-DKD risk (Figures 10B,C). The results showed that the prognostic prediction model effectively stratified patients with T2DM into high-risk and low-risk groups in both the training and validation cohorts. The probability of developing ES-DKD was significantly higher in the high-risk group than in the low-risk group (log-rank *p* < 0.0001).

## Discussion

This study is the first to comprehensively investigate the association between AIP and the risk of ES-DKD in patients with T2DM. Leveraging large-scale real-world data, long-term follow-up results, and SML methods, this study yielded the following key findings. First, AIP was independently associated with the occurrence of ES-DKD in patients with T2DM, exhibiting a linear dose–response relationship. Second, this association remained robust after multivariable adjustment, and subgroup analyses confirmed its broad applicability across populations with diverse clinical characteristics. Third, the predictive value of AIP was further validated using the Boruta algorithm and SML models, ranking fifth in importance among 72 variables. The prognostic prediction model based on the XGBoost algorithm exhibited the best performance, achieving an AUC of 0.752–0.784 for predicting ES-DKD risk in the validation cohort.

Compared with conventional single lipid parameters, AIP integrates information from TG and HDL-C, enabling a more comprehensive reflection of the balance between atherogenic lipid burden and anti-atherogenic protective factors, thereby serving as a more holistic assessment indicator (35). Existing studies across different patient populations have demonstrated that elevated AIP is an important marker of IR and dyslipidemia, and its increased levels (including cumulative exposure) exhibit a linear or nonlinear positive correlation with adverse outcomes of CMD, including cardiovascular disease (11), ischemic stroke (15), MASLD (19), metabolic syndrome (18), diabetes progression (28), and critical cardiovascular disease (36).

Although the predictive value of AIP in the aforementioned CMD has been well established, its application in the field of DKD remains limited. Existing studies have predominantly focused on the middle to late stages of the disease (stage 3 or above) or are based on cross-sectional designs, resulting in a lack of systematic evaluation of the predictive value of AIP during the critical “window of opportunity” of ES-DKD (stages 1–2) (20, 21). To address this evidence gap, the present study analyzed data from 1,006 adult patients with T2DM in a retrospective cohort. The results indicated that baseline AIP is an independent predictor of ES-DKD, consistent with findings from previous studies. These findings further support the clinical potential of AIP as a simple and cost-effective composite biomarker for optimizing early renal risk stratification and guiding individualized primary prevention strategies in patients with T2DM.

Specifically, AIP may promote the development and progression of DKD through the following four pathways. First, the IR pathway. As a reliable surrogate marker of IR, AIP may contribute to glomerular endothelial dysfunction by reducing nitric oxide bioavailability and impairing endothelial function. Meanwhile, hyperinsulinemia can stimulate the expression of insulin-like growth factor-1 and transforming growth factor-*β*, promoting renal fibrosis (37). Second, the lipotoxicity pathway. Elevated AIP levels are often accompanied by increased levels of small dense low-density lipoprotein and oxidized low-density lipoprotein. Under hyperglycemic conditions, these atherogenic lipoproteins are more prone to penetrate the glomerular endothelial barrier and deposit in the mesangial area, inducing mesangial cell proliferation and extracellular matrix expansion, thereby accelerating glomerulosclerosis. Third, the inflammatory pathway. Elevated AIP can activate inflammatory signaling pathways such as nuclear factor-κB, promoting the release of pro-inflammatory cytokines including tumor necrosis factor-*α* and interleukin-6, which further exacerbate inflammatory injury in the glomeruli and tubulointerstitium (38, 39). Fourth, the oxidative stress pathway. Elevated AIP is closely associated with mitochondrial dysfunction and increased production of reactive oxygen species. Oxidative stress can damage renal tubular epithelial cells and podocytes, promoting the development and progression of proteinuria. These four pathways are not independent but rather interact and synergize with each other to drive the progression of DKD from early microalbuminuria to clinical-stage kidney disease (40). In the present study, patients in the high AIP group exhibited higher levels of HbA1c, FBG, and insulin, further supporting the notion that AIP may drive kidney injury by reflecting underlying metabolic disturbances.

In this study, RCS analysis demonstrated that the risk of ES-DKD increased linearly with rising AIP levels, and this association remained stable after adjustment for various confounding factors. This finding suggests that even mildly elevated AIP may signify the initiation of renal microvascular injury. Multivariable Cox regression analyses and subgroup analyses based on potential influencing factors further validated this result, confirming the broad applicability and robustness of the association between AIP and ES-DKD. Kaplan–Meier survival analysis further showed that patients in the highest AIP tertile group had significantly lower long-term event-free survival rates compared with those in the lowest AIP tertile group. These findings are consistent with previous studies on cardiometabolic diseases such as CVD, MASLD, and IR, further supporting the value of AIP in prognostic assessment of ES-DKD in patients with T2DM (11, 19).

It is well recognized that the development and progression of chronic kidney disease in patients with T2DM is not driven by a single factor. As shown in Supplementary Table S2, significant differences were observed between the ES-DKD and non-DKD groups across multiple dimensions, including demographic characteristics, comorbidities, glucose and lipid metabolism indicators, liver and kidney function parameters, and routine blood tests. Although basic statistical methods have preliminarily revealed these differences, they are limited in quantifying the relative importance of each factor within a predictive model. To address this, the present study employed the Boruta algorithm to rank the importance of all candidate variables. The results showed that AIP was identified as an “important feature” in the green region, ranking fifth in importance among all variables, highlighting its central role in ES-DKD risk prediction. Other important features included HbA1c, T3, FBG, GLB, BUN, ALT, GGT, and AST, which collectively reflect the systemic metabolic status of patients with T2DM from aspects such as glycemic control, liver and kidney function, and hormone metabolism. These findings also indirectly reflect the complex interplay between clinical treatment decisions and disease risk (41).

This study systematically evaluated the predictive performance of six prognostic SML models (RSF, Lasso_Cox, CoxBoost, XGBoost, superpc, and plsRcox) for ES-DKD risk. The results showed that most models exhibited good predictive ability, with the exception of superpc, among which the prognostic prediction model based on the XGBoost algorithm demonstrated the best performance, achieving the highest time-dependent AUC. In the training and validation cohorts, this model achieved AUC values of 0.779–0.809 and 0.752–0.784 for predicting 1-year, 3-year, and 5-year ES-DKD risk, respectively. Calibration curves showed that the predicted probabilities were in good agreement with the observed probabilities, and DCA further indicated that the model provided significant clinical net benefit across a wide range of threshold probabilities, suggesting high accuracy and clinical utility. The prognostic prediction models provides clinicians with practical and intuitive risk assessment tools, facilitating more scientific and individualized treatment decisions during the critical intervention window for ES-DKD in patients with T2DM. As demonstrated in studies on cardiovascular and metabolic diseases, the predictive performance of SML algorithms is of great significance for clinicians facing the challenges of early risk stratification and precision management (42).

To translate this simple and low-cost biomarker into clinical practice, this study further developed an online early warning system. Through this platform, clinicians can conveniently input patients’ baseline data and obtain real-time estimates of their future risk of ES-DKD. This evidence-based risk stratification tool enables physicians to initiate intensified lipid-lowering, glucose-lowering, or renoprotective therapies before the onset of clinical DKD (i.e., stages 1–2), thereby seizing the critical opportunity to delay disease progression. This approach holds both clinical utility and health economic value. It should be noted that the conclusions of this study are only applicable to the ES-DKD population and cannot be directly extrapolated to patients with advanced DKD or elderly, long-duration T2DM patients with multiple comorbidities. Furthermore, although this online prediction tool has been publicly released and made freely accessible, its validation is currently based solely on the internal dataset of this study and has not yet been prospectively validated in an independent external cohort. Therefore, this tool should currently be used primarily for research purposes and scientific exploration, and its dissemination and application in real-world clinical settings require further validation.

This study has certain innovations and the following advantages. First, the clinical endpoint focused on ES-DKD (stages 1–2), filling the evidence gap regarding the prognostic value of AIP in ES-DKD. Second, this study comprehensively applied multiple statistical methods, including Cox regression analysis, subgroup analysis, RCS analysis, Kaplan–Meier survival analysis, the Boruta algorithm, cluster heatmaps, and LASSO regression, to systematically investigate the association between AIP and the occurrence of ES-DKD in patients with T2DM from multiple dimensions. The integrated application of these methods enhanced the robustness and credibility of the findings and comprehensively revealed the potential prognostic value of AIP. Furthermore, this study employed six prognostic SML models to predict the occurrence of ES-DKD in patients with T2DM and developed a web-based application to facilitate risk prediction of ES-DKD in clinical practice.

However, several limitations of this study should be considered. First, this study is a single-center retrospective study, with data derived from the hospital information system of one institution, which limits the generalizability of our findings to some extent. In particular, although the six SML prediction models developed in this study performed well in internal validation, the lack of an independent external validation cohort means that their applicability to different geographic regions or ethnic populations requires further investigation. Second, AIP was calculated based solely on baseline values, and dynamic changes in this index during follow-up were not captured. Subsequent studies could collect lipid data at multiple follow-up time points to construct time-dependent models and evaluate the association between dynamic changes in AIP and ES-DKD risk. Third, because the outcome events had already occurred at the start of the study and the exposure factor could not be artificially controlled or randomly assigned, the findings only demonstrate a statistical association between AIP and ES-DKD and cannot infer causality. Fourth, although missing data were handled using random forest imputation (with missing rates all < 10%), imputation itself may introduce certain bias, which may affect the results to some extent. Fifth, patients with baseline eGFR < 60 mL/min/1.73 m^2^ or positive urinary protein were excluded from this study, and thus the conclusions are only applicable to the ES-DKD population. Sixth, missing value imputation in this study was performed before data splitting, which may introduce a minor risk of data leakage. However, given the low missing rates (all variables < 10%) and the robustness of random forest imputation in the context of low missingness, we anticipate that the impact on model performance evaluation is limited. Seventh, this is a retrospective study. The medical records only captured baseline medication use and lacked data on changes in SGLT2 inhibitor and GLP-1 receptor agonist use during follow-up, as well as changes in diet and lifestyle. Therefore, these time-varying confounders could not be adjusted for, which may have some impact on the results. Future prospective studies will improve the collection of relevant follow-up data. Finally, excluding patients with clinical-stage DKD may have introduced selection bias, resulting in the high AIP group being predominantly composed of younger patients with a short disease duration and fewer complications, which can partly explain the counterintuitive baseline characteristics of this group. Therefore, the conclusions of this study are only applicable to the ES-DKD population and cannot be directly extrapolated to patients with advanced DKD or elderly, long-duration T2DM patients with multiple comorbidities.

## Conclusion

This study confirms that AIP is an independent predictor of ES-DKD in patients with T2DM, with a linear positive association between the two. Integrating this low-cost, easily accessible novel composite biomarker into SML frameworks enabled the successful construction of prognostic prediction models that effectively predicts ES-DKD risk in the T2DM population. They may serve as practical tools to assist clinicians in making treatment decisions, facilitating individualized patient management and precise risk stratification. Future multicenter prospective clinical studies are needed to further validate the findings of this study.

### What is already known about this topic?

This study searched relevant literature published up to December 31, 2025, in PubMed and Web of Science without language restrictions, using keywords such as “diabetic kidney disease,” “atherogenic index of plasma,” and “prediction model” in titles and abstracts. The results showed that although several studies on diabetic kidney disease (DKD) prediction exist, most have focused on clinical-stage DKD (stage 3 or above) as the endpoint and are predominantly cross-sectional in design. No study has systematically evaluated the prognostic value of AIP for early-stage DKD (ES-DKD, stages 1–2) in patients with type 2 diabetes mellitus (T2DM), nor has any study reported the construction of supervised machine learning (SML)-based prognostic prediction models incorporating AIP for ES-DKD.

### Key research question

Is AIP an independent risk factor for predicting the occurrence of ES-DKD in patients with T2DM? Can integrating AIP into SML-based prognostic prediction models effectively predict the risk of ES-DKD in the T2DM population?

### Innovation

This study is the first cohort study to systematically investigate the prognostic value of AIP for ES-DKD (stages 1–2) in patients with T2DM. AIP was independently associated with the risk of ES-DKD in a linear dose–response manner. This association remained robust across different clinical subgroups. Furthermore, integrating AIP into SML frameworks successfully yielded high-performance prognostic prediction models, with the XGBoost algorithm demonstrating the best predictive performance (AUC ranging from 0.752 to 0.784 in the validation cohort). To facilitate clinical practice, an online web-based tool was also developed.

### Potential impact on clinical practice

These findings suggest that AIP, as a low-cost and easily accessible biomarker, warrants further exploration for its value in risk stratification of ES-DKD in T2DM patients. The developed prognostic prediction models and online tool provide clinicians with useful instruments for identifying high-risk individuals, enabling the implementation of personalized primary prevention strategies and early interventions during this critical window of disease progression.

## Data Availability

The raw data supporting the conclusions of this article will be made available by the authors, without undue reservation.

## Footnotes

1
https://www.medsta.cn/software

2
https://earlystagedkdriskprediction.shinyapps.io/DynNomapp/
