# Supplementary material for: Prognostic value of the atherogenic index of plasma for early-stage diabetic kidney disease in type 2 diabetes: a retrospective cohort study using supervised machine learning
Source: Front Nutr. 2026 Jun 30;13:1844510. doi: 10.3389/fnut.2026.1844510 (PMC13377558; doi:10.3389/fnut.2026.1844510)
Supplement: Supplementary file 1 [file Supplementary_file_1.docx]

**Supplementary Materials**

**
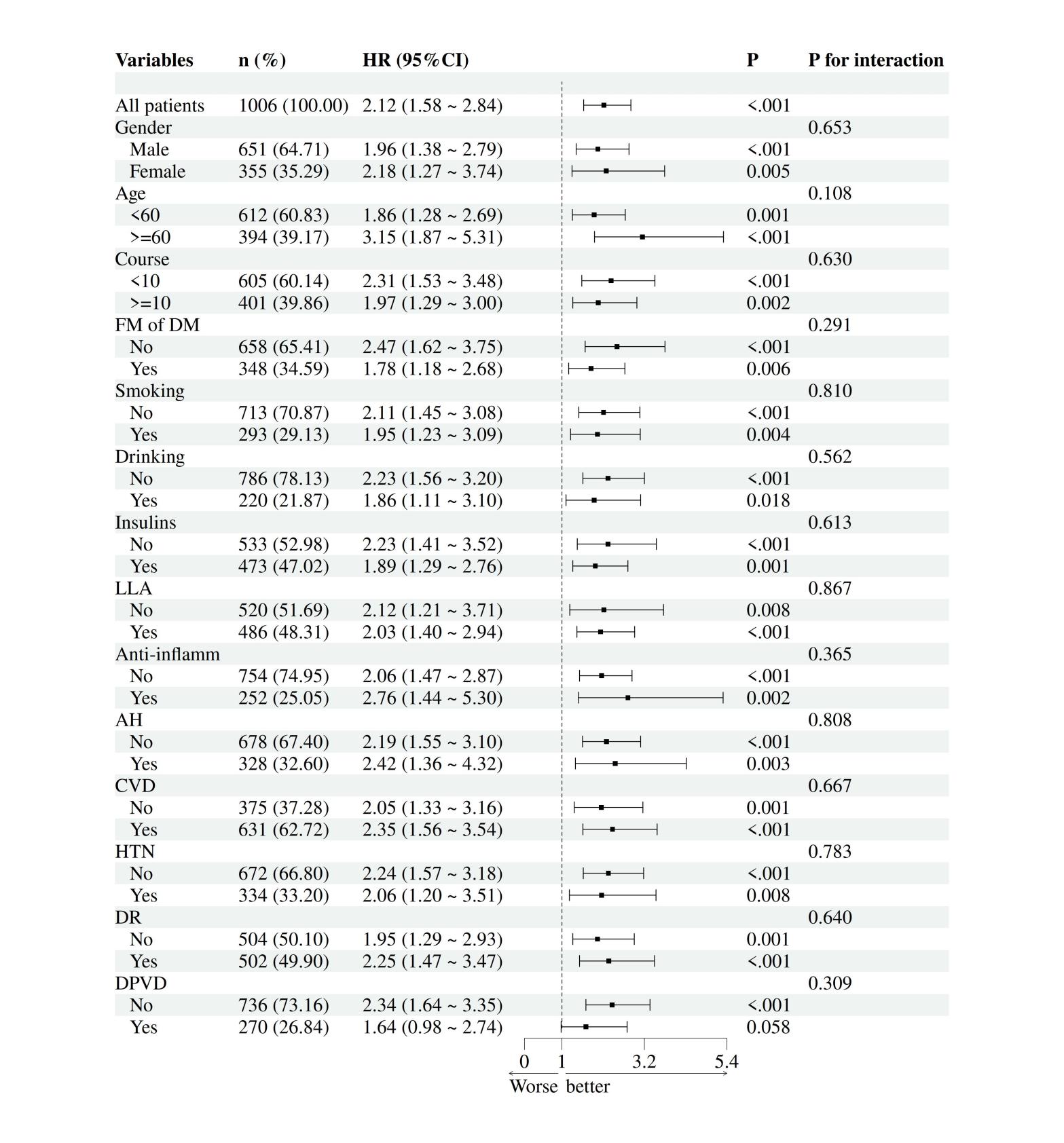
**

**Fig. S1** Cox proportional hazards regression and subgroup analysis forest plot for the association of atherogenic index of plasma (AIP) with early-stage diabetic kidney disease (ES-DKD) risk (unadjusted). *Abbreviation*: HR, hazard ratio; CI, confidence interval; FM of DM, family history of diabetes mellitus; LLA, lipid-lowering agents; Anti-inflamm, anti-inflammatory agents; AH, antihypertensive agents; CVD, cardiovascular disease; HTN, hypertension; DR, diabetic retinopathy; DPVD, diabetic peripheral vascular disease.

**
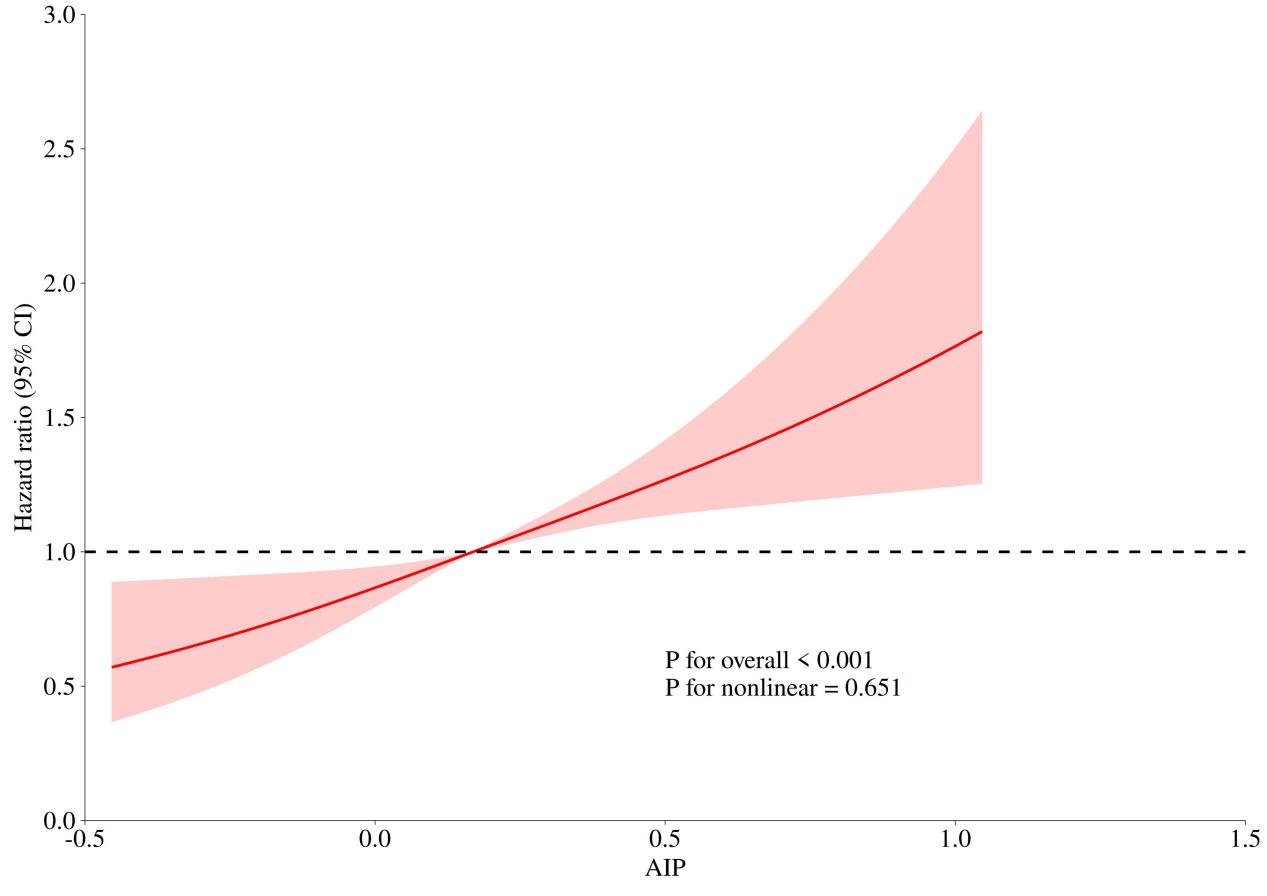
**

**Fig. S2** Restricted cubic spline (RCS) analysis for the association between atherogenic index of plasma (AIP) and early-stage diabetic kidney disease (ES-DKD) risk (unadjusted). The RCS curve demonstrates the dose-response relationship between AIP as a continuous variable and the risk of ES-DKD.

**
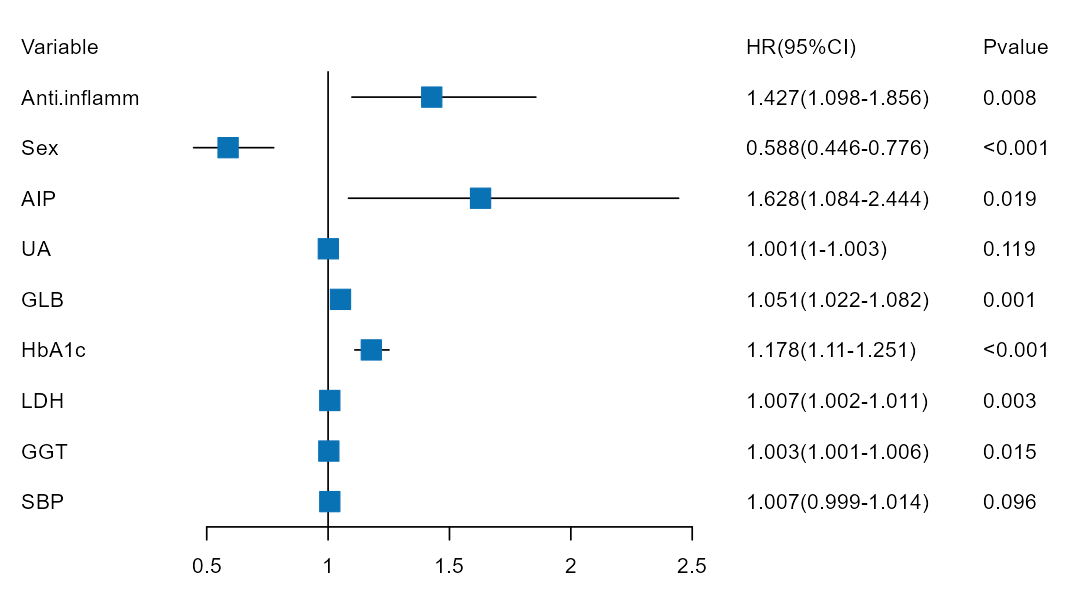
Fig. S3** Multivariable Cox regression analysis of the final selected predictors. *Abbreviation*: AIP, atherogenic index of plasma; Anti.inflamm, anti-inflammatory agents; GGT, gamma-glutamyl transferase; GLB, globulin; HbA1c, glycated hemoglobin; LDH, lactate dehydrogenase; SBP, systolic blood pressure; UA, uric acid.

**
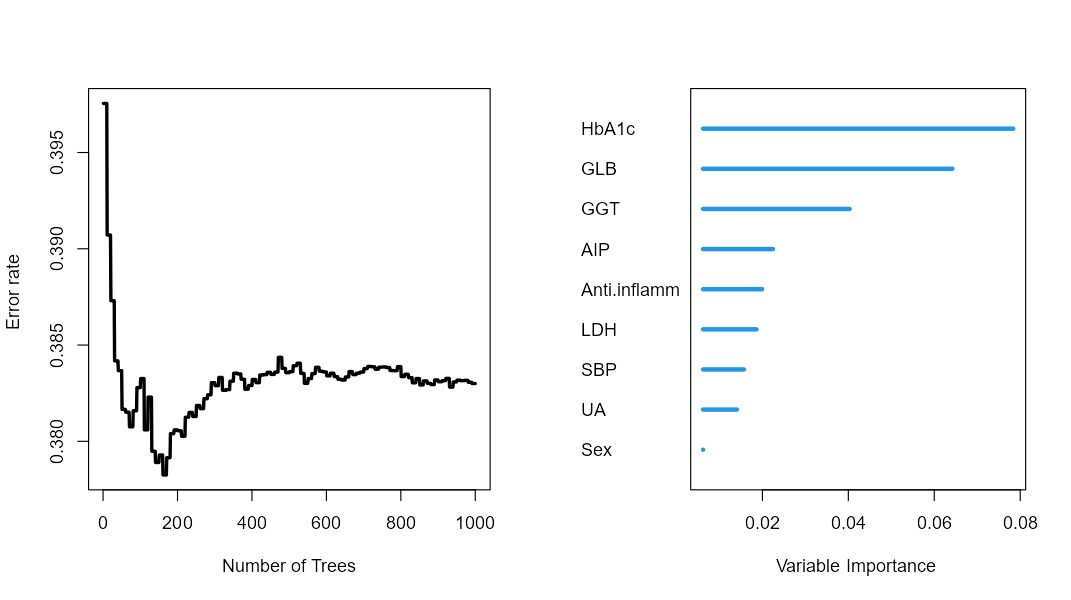
**

**Fig. S4** Variable importance ranking from the random survival forest (RSF) model. *Abbreviation*: AIP, atherogenic index of plasma; Anti.inflamm, anti-inflammatory agents; GGT, gamma-glutamyl transferase; GLB, globulin; HbA1c, glycated hemoglobin; LDH, lactate dehydrogenase; SBP, systolic blood pressure; UA, uric acid.

**
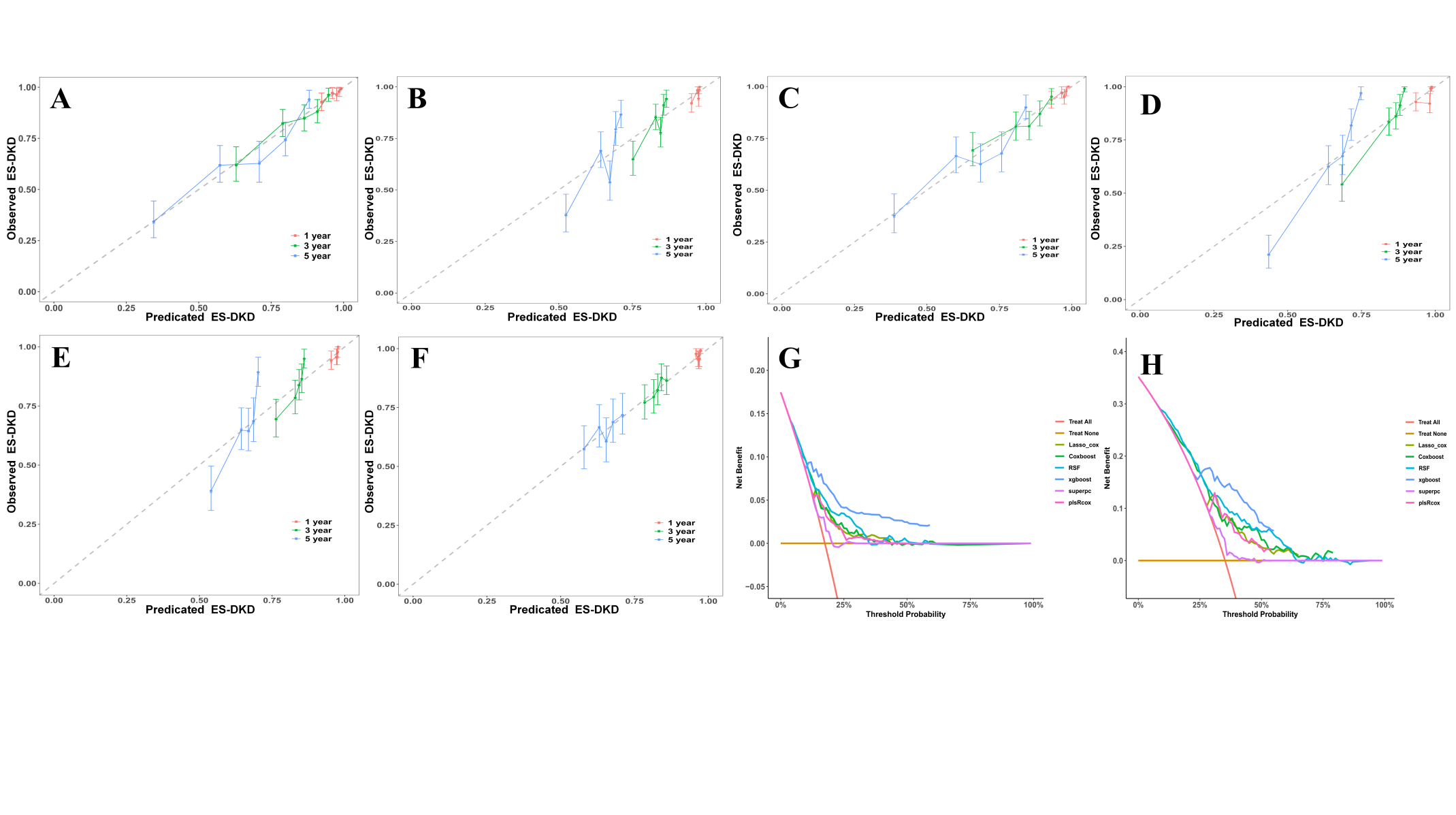
**

**Fig. S5** Calibration curves and decision curve analysis of six prognostic machine learning models for predicting early-stage diabetic kidney disease (ES-DKD) in the training cohort. **A-F.** Calibration curves for RSF, Lasso_Cox, coxboost, XGBoost, superpc, and plsRcox models at 1 year, 3 years, and 5 years of follow-up, respectively. The x-axis represents the predicted probability of ES-DKD, and the y-axis represents the observed probability. The diagonal dashed line indicates perfect calibration, where predicted probabilities equal observed probabilities. The solid curves represent the actual performance of the models, with closer proximity to the diagonal line indicating better calibration. **G-H.** Decision curve analysis of the six machine learning models at 3 years and 5 years of follow-up. The x-axis represents the threshold probability, and the y-axis represents the net benefit. The horizontal line represents the assumption that no patients have ES-DKD (intervention for none), and the diagonal line represents the assumption that all patients have ES-DKD (intervention for all). *Abbreviation*: RSF, random survival forest; Lasso_Cox, lasso-penalized Cox proportional hazards model; XGBoost, extreme gradient boosting; superpc, supervised principal components; plsRcox, partial least squares regression for Cox model.


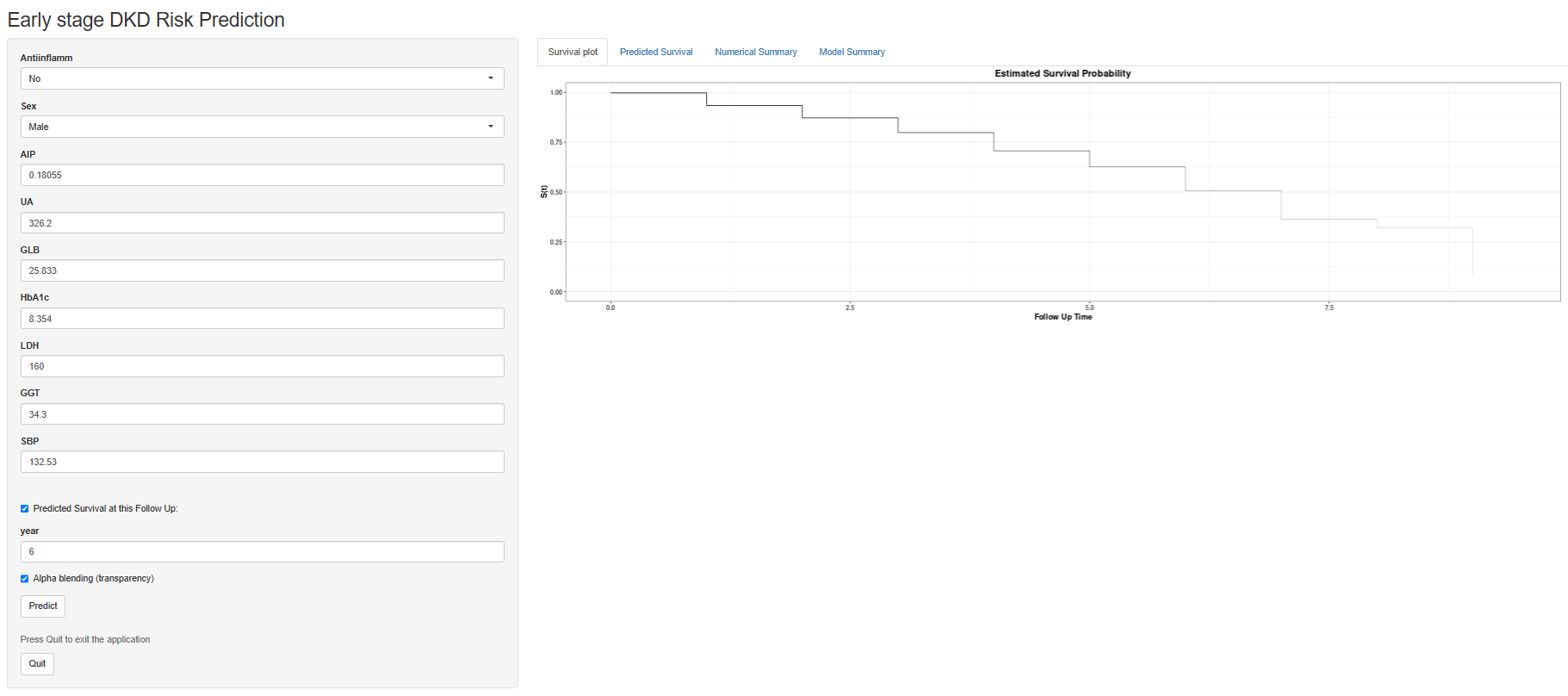


**Fig. S6** Web-based online tool for predicting early-stage diabetic kidney disease (ES-DKD) risk. The web application allows clinicians to input baseline patient parameters and obtain individualized risk probabilities for ES-DKD at 1 year, 3 years, and 5 years. This user-friendly tool facilitates the integration of the prognostic prediction model into clinical practice, supporting personalized risk assessment and decision-making. The online tool is available at: <https://earlystagedkdriskprediction.shinyapps.io/DynNomapp/>

**Table S1** Percentage of missing data for all variables included in the study.

| Variables | Missing data (%) |
| --- | --- |
| **Demographics** |  |
| Age (years) | 0.00 |
| Male, n (%) | 0.00 |
| Ethnicity-Han, n (%) | 0.00 |
| Married, n (%) | 0.00 |
| Career-Business/Service, n (%) | 0.00 |
| **Comorbidities** |  |
| HTN, n (%) | 0.00 |
| MASLD, n (%) | 0.00 |
| CVD, n (%) | 0.00 |
| DR, n (%) | 0.00 |
| DSPN, n (%) | 0.00 |
| DPVD, n (%) | 0.00 |
| **Lifestyle and FM** |  |
| Smoking, n (%) | 0.00 |
| Drinking, n (%) | 0.00 |
| FM of DM, n (%) | 0.00 |
| FM of HTN, n (%) | 0.00 |
| **Medications** |  |
| Insulins, n (%) | 0.00 |
| OHA, n (%) | 0.00 |
| LLA, n (%) | 0.00 |
| Anti-inflamm, n (%) | 0.00 |
| Renoprot, n (%) | 0.00 |
| AH, n (%) | 0.00 |
| **Clinical indicators** |  |
| Course (years) | 0.00 |
| SBP (mmHg) | 0.00 |
| DBP (mmHg) | 0.00 |
| BMI (kg/m2) | 0.00 |
| **Biochemical indicators** |  |
| Glucose metabolic indices |  |
| HbA1c (%) | 1.79 |
| FBG (mmo/L) | 0.00 |
| FINS (mU/L) | 1.79 |
| FCP (ng/mL) | 2.78 |
| 2hBG (mmo/L) | 1.99 |
| 2hINS (mU/L) | 1.99 |
| 2hCP (ng/mL) | 2.98 |
| Liver function tests |  |
| AST (U/L) | 0.00 |
| ALT (U/L) | 0.00 |
| TB (umol/L) | 0.00 |
| DB (umol/L) | 0.00 |
| IB (umol/L) | 0.00 |
| TP (g/L) | 0.00 |
| ALB (g/L) | 0.00 |
| GLB (g/L) | 0.00 |
| ALP (U/L) | 0.30 |
| GGT (U/L) | 0.30 |
| CHE (ku/L) | 0.30 |
| TBA (μmol/L) | 0.30 |
| LDH (U/L) | 0.30 |
| Renal function tests |  |
| BUN (mmol/L) | 0.00 |
| SCr (umol/L) | 0.00 |
| eGFR [ml/(min·1.73m2)] | 0.00 |
| UA (umol/L) | 0.00 |
| Serum electrolytes |  |
| Potassium (mmo/L) | 0.00 |
| Sodium (mmo/L) | 0.00 |
| Chloride (mmo/L) | 0.00 |
| Calcium (mmo/L) | 0.00 |
| Phosphate (mmo/L) | 0.20 |
| Lipid profile |  |
| TC (mmo/L) | 0.00 |
| TG (mmo/L) | 0.00 |
| HDL (mmo/L) | 0.00 |
| LDL (mmo/L) | 0.00 |
| **Hormones** |  |
| T3 (ng/ml) | 6.56 |
| T4 (ug/dL) | 6.46 |
| TSH (uIU/mL) | 6.16 |
| 25(OH)D (nmol L) | 3.78 |
| **Complete blood count** |  |
| WBC (10^9/L) | 0.70 |
| RBC (10^12/L) | 0.70 |
| Hb (g/L) | 0.70 |
| Plt (10^9/L) | 0.70 |
| LYMPH (10^9/L) | 0.70 |
| MONO (10^9/L) | 0.70 |
| NEUT (10^9/L) | 0.70 |
| EO (10^9/L) | 0.70 |
| BASO (10^9/L) | 0.70 |

*Abbreviation*: HTN, hypertension; MASLD, metabolic dysfunction-associated steatotic liver disease; CVD, cardiovascular disease; DR, diabetic retinopathy; DSPN, diabetic sensorimotor polyneuropathy; DPVD, diabetic peripheral vascular disease; FM, family history; DM, diabetes mellitus; OHA, oral hypoglycemic agents; LLA, lipid-lowering agents; Anti-inflamm, anti-inflammatory agents; Renoprot, renoprotective agents; AH, antihypertensive agents; SBP, systolic blood pressure; DBP, diastolic blood pressure; BMI, body mass index; HbA1c, glycated hemoglobin; FBG, fasting blood glucose; FINS, fasting insulin; FCP, fasting C-peptide; 2hBG, 2-hour postprandial blood glucose; 2hINS, 2-hour postprandial insulin; 2hCP, 2-hour postprandial C-peptide; AST, aspartate aminotransferase; ALT, alanine aminotransferase; TB, total bilirubin; DB, direct bilirubin; IB, indirect bilirubin; TP, total protein; ALB, albumin; GLB, globulin; ALP, alkaline phosphatase; GGT, gamma-glutamyl transferase; CHE, cholinesterase; TBA, total bile acids; LDH, lactate dehydrogenase; BUN, blood urea nitrogen; SCr, serum creatinine; eGFR, estimated glomerular filtration rate; UA, uric acid; TC, total cholesterol; TG, triglycerides; HDL, high-density lipoprotein; LDL, low-density lipoprotein; T3, triiodothyronine; T4, thyroxine; TSH, thyroid-stimulating hormone; 25(OH)D, 25-hydroxyvitamin D; WBC, white blood cell; RBC, red blood cell; Hb, hemoglobin; Plt, platelet; LYMPH, lymphocyte; MONO, monocyte; NEUT, neutrophil; EO, eosinophil; BASO, basophil.

**Table S2** Baseline characteristics of Non-DKD and early-stage DKD

| Characteristics | Non-DKD | Early-stage DKD | P-value |
| --- | --- | --- | --- |
|  | (n=602) | (n=404) |  |
| **Demographics** |  |  |  |
| Age (years) | 56.77 ± 9.71 | 56.17±10.38 | 0.348 |
| Male, n (%) | 374 (62.13) | 277 (68.56) | **0.036** |
| Ethnicity-Han, n (%) | 575 (95.51) | 379 (93.81) | 0.232 |
| Married, n (%) | 586 (97.34) | 393 (97.28) | 0.950 |
| Career-Business/Service, n (%) | 439 (72.92) | 265 (65.59) | **0.002** |
| **Comorbidities** |  |  |  |
| HTN, n (%) | 188 (31.23) | 146 (36.14) | 0.105 |
| MASLD, n (%) | 277 (46.01) | 209 (51.73) | 0.075 |
| CVD, n (%) | 374 (62.13) | 256 (63.37) | 0.690 |
| DR, n (%) | 306 (50.83) | 196 (48.51) | 0.471 |
| DSPN, n (%) | 490 (81.40) | 333 (82.43) | 0.678 |
| DPVD, n (%) | 166 (27.57) | 104 (25.74) | 0.520 |
| **Lifestyle and FM** |  |  |  |
| Smoking, n (%) | 158 (26.25) | 135 (33.42) | **0.014** |
| Drinking, n (%) | 123 (20.43) | 97 (24.01) | 0.178 |
| FM of DM, n (%) | 193 (32.06) | 155 (38.37) | **0.039** |
| FM of HTN, n (%) | 94 (15.61) | 62 (15.35) | 0.908 |
| **Medications** |  |  |  |
| Insulins, n (%) | 246 (40.86) | 227 (56.19) | **<0.001** |
| OHA, n (%) | 593 (98.50) | 398 (98.51) | 0.990 |
| LLA, n (%) | 276 (45.85) | 210 (51.98) | 0.056 |
| Anti-inflamm, n (%) | 134 (22.26) | 118 (29.21) | **0.013** |
| Renoprot, n (%) | 8 (1.33) | 16 (3.96) | **0.007** |
| AH, n (%) | 181 (30.07) | 147 (36.39) | **0.036** |
| **Clinical indicators** |  |  |  |
| Course (years) | 7.00 (3.00, 12.00) | 8.00 (3.00, 12.00) | 0.568 |
| SBP (mmHg) | 130.00 (120.00, 140.75) | 132.00 (123.00, 144.25) | **0.022** |
| DBP (mmHg) | 78.00 (72.00, 84.00) | 78.00 (74.00, 86.00) | **0.032** |
| BMI (kg/m2) | 24.01 (22.13, 25.89) | 24.68 (22.60, 26.56) | **0.001** |
| **Biochemical indicators** |  |  |  |
| Glucose metabolic indices |  |  |  |
| HbA1c (%) | 7.60 (6.70, 9.20) | 8.40 (7.10, 10.20) | **<0.001** |
| FBG (mmo/L) | 8.20 (6.90, 10.20) | 9.20 (7.57, 11.72) | **<0.001** |
| FINS (mU/L) | 7.16 (4.93, 10.20) | 7.79 (5.33, 10.55) | 0.057 |
| FCP (ng/mL) | 1.39 (1.03, 1.79) | 1.45 (1.09, 1.93) | **0.035** |
| 2hBG (mmo/L) | 16.90 (13.50, 20.10) | 18.00 (14.97, 21.34) | **<0.001** |
| 2hINS (mU/L) | 26.44 (17.48, 42.20) | 25.15 (15.62, 37.91) | 0.094 |
| 2hCP (ng/mL) | 3.90 (2.89, 5.75) | 3.75 (2.64, 5.29) | **0.045** |
| Liver function tests |  |  |  |
| AST (U/L) | 19.00 (16.00, 23.00) | 21.00 (16.00, 27.25) | **<0.001** |
| ALT (U/L) | 20.00 (15.00, 27.00) | 24.00 (16.00, 35.00) | **<0.001** |
| TB (umol/L) | 14.40 (11.40, 18.00) | 14.70 (11.40, 18.80) | 0.448 |
| DB (umol/L) | 2.80 (2.10, 3.50) | 2.80 (2.10, 3.50) | 0.986 |
| IB (umol/L) | 11.70 (9.20, 14.60) | 11.95 (9.00, 15.20) | 0.367 |
| TP (g/L) | 68.45 (63.80, 72.20) | 70.00 (66.00, 73.93) | **<0.001** |
| ALB (g/L) | 43.30 (41.12, 45.40) | 43.50 (41.50, 45.40) | 0.515 |
| GLB (g/L) | 24.90 (22.33, 27.80) | 26.30 (23.60, 30.00) | **<0.001** |
| ALP (U/L) | 84.66 (68.00, 102.00) | 88.50 (71.00, 107.00) | **0.020** |
| GGT (U/L) | 23.40 (17.40, 33.23) | 28.15 (20.80, 43.55) | **<0.001** |
| CHE (ku/L) | 8.25 (7.38, 9.25) | 8.37 (7.42, 9.42) | 0.404 |
| TBA (μmol/L) | 4.00 (2.60, 6.00) | 4.30 (2.77, 6.60) | **0.037** |
| LDH (U/L) | 156.00 (138.00, 173.75) | 162.50 (138.75, 183.00) | **0.045** |
| Renal function tests |  |  |  |
| BUN (mmol/L) | 5.44 (4.58, 6.28) | 5.42 (4.58, 6.47) | 0.471 |
| SCr (umol/L) | 65.00 (57.00, 73.00) | 65.00 (56.00, 75.00) | 0.664 |
| eGFR [ml/(min·1.73m^2^)] | 99.19 (93.19, 106.02) | 100.79 (92.98, 108.57) | 0.114 |
| UA (umol/L) | 310.50 (266.00, 365.00) | 336.00 (285.00, 380.25) | **<0.001** |
| Serum electrolytes |  |  |  |
| Potassium (mmo/L) | 3.95 (3.72, 4.16) | 3.93 (3.72, 4.11) | 0.422 |
| Sodium (mmo/L) | 140.00 (138.00, 141.00) | 139.00 (138.00, 141.00) | **<0.001** |
| Chloride (mmo/L) | 105.00 (104.00, 107.00) | 105.00 (103.00, 106.00) | **<0.001** |
| Calcium (mmo/L) | 2.26 (2.18, 2.33) | 2.27 (2.19, 2.35) | 0.052 |
| Phosphate (mmo/L) | 1.18 (1.07, 1.30) | 1.18 (1.06, 1.29) | 0.550 |
| Lipid profile |  |  |  |
| TC (mmo/L) | 4.39 (3.77, 4.95) | 4.46 (3.83, 5.19) | 0.063 |
| TG (mmo/L) | 1.39 (1.01, 1.96) | 1.66 (1.17, 2.56) | **<0.001** |
| HDL (mmo/L) | 1.06 (0.90, 1.20) | 0.97 (0.85, 1.15) | **<0.001** |
| LDL (mmo/L) | 2.80 (2.29, 3.21) | 2.83 (2.38, 3.40) | 0.083 |
| **Hormones** |  |  |  |
| T3 (ng/ml) | 0.98 (0.87, 1.11) | 0.97 (0.85, 1.11) | 0.307 |
| T4 (ug/dL) | 7.38 (6.35, 8.54) | 7.31 (6.25, 8.66) | 0.897 |
| TSH (uIU/mL) | 2.38 (1.55, 3.62) | 2.33 (1.36, 3.58) | 0.282 |
| 25(OH)D (nmol L) | 15.75 (11.08, 19.75) | 14.31 (10.86, 19.04) | 0.055 |
| **Complete blood count** |  |  |  |
| WBC (10^9/L) | 5.72 (4.91, 6.65) | 5.90 (5.04, 7.00) | **0.015** |
| RBC (10^12/L) | 4.83 (4.56, 5.15) | 4.91 (4.63, 5.27) | **0.009** |
| Hb (g/L) | 148.50 (139.00, 158.00) | 153.00 (141.00, 160.00) | **0.008** |
| Plt (10^9/L) | 177.00 (150.00, 220.75) | 174.50 (142.00, 223.00) | 0.475 |
| LYMPH (10^9/L) | 1.90 (1.56, 2.40) | 2.00 (1.58, 2.40) | 0.204 |
| MONO (10^9/L) | 0.36 (0.30, 0.44) | 0.39 (0.30, 0.49) | 0.099 |
| NEUT (10^9/L) | 3.20 (2.60, 3.90) | 3.40 (2.70, 4.10) | **0.006** |
| EO (10^9/L) | 0.10 (0.08, 0.20) | 0.11 (0.10, 0.20) | **0.026** |
| BASO (10^9/L) | 0.01 (0.00, 0.02) | 0.01 (0.00, 0.03) | 0.315 |
| **AIP** | 0.13 (-0.04, 0.31) | 0.23 (0.04, 0.45) | **<0.001** |

*Abbreviation:* DKD, diabetic kidney disease; ES-DKD, early-stage diabetic kidney disease; HTN, hypertension; MASLD, metabolic dysfunction-associated steatotic liver disease; CVD, cardiovascular disease; DR, diabetic retinopathy; DSPN, diabetic sensorimotor polyneuropathy; DPVD, diabetic peripheral vascular disease; FM, family history; DM, diabetes mellitus; OHA, oral hypoglycemic agents; LLA, lipid-lowering agents; Anti-inflamm, anti-inflammatory agents; Renoprot, renoprotective agents; AH, antihypertensive agents; SBP, systolic blood pressure; DBP, diastolic blood pressure; BMI, body mass index; HbA1c, glycated hemoglobin; FBG, fasting blood glucose; FINS, fasting insulin; FCP, fasting C-peptide; 2hBG, 2-hour postprandial blood glucose; 2hINS, 2-hour postprandial insulin; 2hCP, 2-hour postprandial C-peptide; AST, aspartate aminotransferase; ALT, alanine aminotransferase; TB, total bilirubin; DB, direct bilirubin; IB, indirect bilirubin; TP, total protein; ALB, albumin; GLB, globulin; ALP, alkaline phosphatase; GGT, gamma-glutamyl transferase; CHE, cholinesterase; TBA, total bile acids; LDH, lactate dehydrogenase; BUN, blood urea nitrogen; SCr, serum creatinine; eGFR, estimated glomerular filtration rate; UA, uric acid; TC, total cholesterol; TG, triglycerides; HDL, high-density lipoprotein; LDL, low-density lipoprotein; T3, triiodothyronine; T4, thyroxine; TSH, thyroid-stimulating hormone; 25(OH)D, 25-hydroxyvitamin D; WBC, white blood cell; RBC, red blood cell; Hb, hemoglobin; Plt, platelet; LYMPH, lymphocyte; MONO, monocyte; NEUT, neutrophil; EO, eosinophil; BASO, basophil; AIP, atherogenic index of plasma.

**Table S3** Subgroup analysis on Cox proportional hazards regression according to the other potential risk factors

|  | **Unadjusted** | | | | **Adjusted** | | | |
| --- | --- | --- | --- | --- | --- | --- | --- | --- |
| **Variables** | **n (%)** | **HR (95% CI)** | ***P*** | ***P* for interaction** | **n (%)** | **HR (95% CI)** | ***P*** | ***P* for interaction** |
| All patients | 1006 (100.00) | 2.12 (1.58 ~ 2.84) | **<0.001** |  | 1006 (100.00) | 1.96 (1.41 ~ 2.72) | **<0.001** |  |
| Ethnicity-Han |  |  |  | 0.186 |  |  |  | 0.174 |
| No | 52 (5.17) | 0.85 (0.22 ~ 3.28) | 0.812 |  | 52 (5.17) | 0.08 (0.01 ~ 1.06) | 0.055 |  |
| Yes | 954 (94.83) | 2.21 (1.64 ~ 2.98) | **<0.001** |  | 954 (94.83) | 2.01 (1.43 ~ 2.82) | **<0.001** |  |
| Business/Service |  |  |  | 0.024 |  |  |  | 0.017 |
| No | 302 (30.02) | 1.21 (0.68 ~ 2.17) | 0.521 |  | 302 (30.02) | 1.15 (0.61 ~ 2.14) | 0.666 |  |
| Yes | 704 (69.98) | 2.56 (1.83 ~ 3.60) | **<0.001** |  | 704 (69.98) | 2.47 (1.68 ~ 3.62) | **<0.001** |  |
| FM of HTN |  |  |  | 0.310 |  |  |  | 0.473 |
| No | 850 (84.49) | 2.36 (1.69 ~ 3.31) | **<0.001** |  | 850 (84.49) | 2.10 (1.45 ~ 3.04) | **<0.001** |  |
| Yes | 156 (15.51) | 1.64 (0.86 ~ 3.11) | 0.131 |  | 156 (15.51) | 1.79 (0.70 ~ 4.54) | 0.222 |  |
| OHA |  |  |  | 0.309 |  |  |  | 0.677 |
| No | 15 (1.49) | 0.13 (0.00 ~ 9.48) | 0.349 |  | 15 (1.49) | 0.00 (0.00 ~ Inf) | 0.996 |  |
| Yes | 991 (98.51) | 2.15 (1.60 ~ 2.88) | **<0.001** |  | 991 (98.51) | 1.99 (1.43 ~ 2.76) | **<0.001** |  |
| HbA1c |  |  |  | 0.070 |  |  |  | 0.083 |
| <7 | 282 (28.03) | 3.15 (1.69 ~ 5.85) | **<0.001** |  | 282 (28.03) | 3.47 (1.58 ~ 7.60) | **0.002** |  |
| >=7 | 724 (71.97) | 1.69 (1.21 ~ 2.37) | **0.002** |  | 724 (71.97) | 1.66 (1.15 ~ 2.39) | **0.007** |  |
| DSPN |  |  |  | 0.947 |  |  |  | 0.722 |
| No | 183 (18.19) | 2.05 (0.93 ~ 4.49) | 0.074 |  | 183 (18.19) | 1.30 (0.48 ~ 3.47) | 0.606 |  |
| Yes | 823 (81.81) | 2.13 (1.56 ~ 2.92) | **<0.001** |  | 823 (81.81) | 2.13 (1.49 ~ 3.06) | **<0.001** |  |
| MASLD |  |  |  | 0.307 |  |  |  | 0.375 |
| No | 520 (51.69) | 2.51 (1.60 ~ 3.95) | **<0.001** |  | 520 (51.69) | 2.59 (1.56 ~ 4.31) | **<0.001** |  |
| Yes | 486 (48.31) | 1.83 (1.20 ~ 2.77) | **0.005** |  | 486 (48.31) | 1.43 (0.90 ~ 2.28) | 0.127 |  |

*Abbreviation:* HR, hazard ratio; CI, confidence interval; FM, family history; HTN, hypertension; OHA, oral hypoglycemic agents; HbA1c, glycated hemoglobin; DSPN, diabetic sensorimotor polyneuropathy; MASLD, metabolic dysfunction-associated steatotic liver disease.

**Table S4** Baseline characteristics of the training and validation cohorts stratified by early-stage diabetic kidney disease (ES-DKD) status

| Characteristics | Training cohort (n=704) | | |  | Validation cohort (n=302) | | | *P* |
| --- | --- | --- | --- | --- | --- | --- | --- | --- |
|  | Non-DKD (n=427) | DKD (n=277) | *P* |  | Non-DKD (n=175) | DKD (n=127) | *P* |  |
| **Demographics** |  |  |  |  |  |  |  |  |
| Age (years) | 57.00 (51.00, 64.00) | 56.00 (51.00, 63.00) | 0.263 |  | 56.00 (50.00, 62.00) | 56.00 (50.00, 61.00) | 0.894 | 0.142 |
| Male, n (%) | 260 (60.89) | 193 (69.68) | **0.017** |  | 114 (65.14) | 84 (66.14) | 0.857 | 0.711 |
| Ethnicity-Han, n (%) | 405 (94.85) | 261 (94.22) | 0.720 |  | 170 (97.14) | 118 (92.91) | 0.084 | 0.617 |
| Married, n (%) | 419 (98.13) | 273 (98.56) | 0.895 |  | 167 (95.43) | 120 (94.49) | 0.710 | **0.003** |
| Career-Business/Service, n (%) | 315 (73.77) | 185 (66.79) | 0.061 |  | 124 (70.86) | 80 (62.99) | **0.046** | 0.614 |
| **Comorbidities** |  |  |  |  |  |  |  |  |
| HTN, n (%) | 140 (32.79) | 93 (33.57) | 0.828 |  | 48 (27.43) | 53 (41.73) | **0.009** | 0.915 |
| MASLD, n (%) | 191 (44.73) | 142 (51.26) | 0.090 |  | 86 (49.14) | 67 (52.76) | 0.535 | 0.657 |
| CVD, n (%) | 277 (64.87) | 172 (62.09) | 0.454 |  | 97 (55.43) | 84 (66.14) | 0.061 | 0.690 |
| DR, n (%) | 213 (49.88) | 140 (50.54) | 0.864 |  | 93 (53.14) | 56 (44.09) | 0.121 | 0.815 |
| DSPN, n (%) | 349 (81.73) | 225 (81.23) | 0.866 |  | 141 (80.57) | 108 (85.04) | 0.314 | 0.730 |
| DPVD, n (%) | 110 (25.76) | 71 (25.63) | 0.969 |  | 56 (32.00) | 33 (25.98) | 0.258 | 0.217 |
| **Lifestyle and FM** |  |  |  |  |  |  |  |  |
| Smoking, n (%) | 114 (26.70) | 85 (30.69) | 0.251 |  | 44 (25.14) | 50 (39.37) | **0.008** | 0.360 |
| Drinking, n (%) | 89 (20.84) | 60 (21.66) | 0.795 |  | 34 (19.43) | 37 (29.13) | 0.050 | 0.409 |
| FM of DM, n (%) | 133 (31.15) | 105 (37.91) | 0.064 |  | 60 (34.29) | 50 (39.37) | 0.365 | 0.424 |
| FM of HTN, n (%) | 125 (29.27) | 93 (33.57) | 0.228 |  | 25 (14.29) | 21 (16.54) | 0.591 | 0.875 |
| **Medications** |  |  |  |  |  |  |  |  |
| Insulins, n (%) | 183 (42.86) | 152 (54.87) | **0.002** |  | 63 (36.00) | 75 (59.06) | **<0.001** | 0.582 |
| OHA, n (%) | 420 (98.36) | 274 (98.92) | 0.777 |  | 173 (98.86) | 124 (97.64) | 0.717 | 1.000 |
| LLA, n (%) | 205 (48.01) | 142 (51.26) | 0.399 |  | 71 (40.57) | 68 (53.54) | **0.026** | 0.342 |
| Anti-inflamm, n (%) | 99 (23.19) | 83 (29.96) | **0.045** |  | 35 (20.00) | 35 (27.56) | 0.124 | 0.370 |
| Renoprot, n (%) | 4 (0.94) | 10 (3.61) | **0.013** |  | 4 (2.29) | 6 (4.72) | 0.399 | 0.208 |
| AH, n (%) | 129 (30.21) | 94 (33.94) | 0.299 |  | 52 (29.71) | 53 (41.73) | **0.030** | 0.338 |
| **Clinical indicators** |  |  |  |  |  |  |  |  |
| Course (years) | 7.00 (3.00, 12.00) | 7.00 (3.00, 12.00) | 0.682 |  | 7.00 (2.00, 12.00) | 8.00 (4.00, 11.50) | 0.738 | 0.586 |
| SBP (mmHg) | 129.00 (119.00, 141.00) | 133.00 (125.00, 145.00) | **0.002** |  | 132.00 (123.00, 139.50) | 130.00 (119.00, 141.00) | 0.577 | 0.707 |
| DBP (mmHg) | 77.00 (71.00, 83.00) | 78.00 (74.00, 87.00) | **0.005** |  | 79.00 (73.50, 86.00) | 78.00 (73.50, 85.00) | 0.499 | 0.215 |
| BMI (kg/m2) | 24.00 (22.23, 25.85) | 24.77 (22.60, 26.67) | **0.002** |  | 24.03 (21.97, 25.95) | 24.09 (22.59, 26.21) | 0.239 | 0.529 |
| **Biochemical indicators** |  |  |  |  |  |  |  |  |
| Glucose metabolic indices |  |  |  |  |  |  |  |  |
| HbA1c (%) | 7.60 (6.70, 9.20) | 8.40 (7.10, 10.20) | **<0.001** |  | 7.70 (6.65, 9.10) | 8.30 (7.25, 10.20) | **<0.001** | 0.668 |
| FBG (mmo/L) | 8.20 (6.80, 10.10) | 9.30 (7.60, 11.80) | **<0.001** |  | 8.30 (7.00, 10.50) | 8.80 (7.40, 11.14) | **0.014** | 0.724 |
| FINS (mU/L) | 7.18 (4.93, 10.11) | 8.10 (5.26, 11.05) | **0.043** |  | 7.11 (5.01, 11.03) | 7.15 (5.56, 10.09) | 0.694 | 0.701 |
| FCP (ng/mL) | 1.39 (1.02, 1.77) | 1.46 (1.09, 1.97) | **0.015** |  | 1.41 (1.05, 1.90) | 1.44 (1.04, 1.83) | 0.967 | 0.689 |
| 2hBG (mmo/L) | 16.90 (13.30, 20.10) | 18.30 (15.00, 21.80) | **<0.001** |  | 16.90 (13.60, 20.00) | 17.50 (14.70, 20.15) | 0.277 | 0.576 |
| 2hINS (mU/L) | 26.06 (17.77, 41.77) | 25.65 (16.33, 38.14) | 0.358 |  | 27.39 (16.66, 42.50) | 24.53 (14.84, 36.31) | 0.096 | 0.827 |
| 2hCP (ng/mL) | 3.95 (2.87, 5.76) | 3.78 (2.65, 5.39) | 0.091 |  | 3.75 (2.93, 5.63) | 3.73 (2.63, 5.19) | 0.279 | 0.885 |
| Liver function tests |  |  |  |  |  |  |  |  |
| AST (U/L) | 19.00 (16.00, 23.00) | 21.00 (16.00, 28.00) | **<0.001** |  | 19.00 (16.00, 24.00) | 21.00 (16.00, 26.50) | 0.068 | 0.758 |
| ALT (U/L) | 20.00 (15.00, 28.00) | 24.00 (16.00, 37.00) | **<0.001** |  | 20.00 (16.00, 26.00) | 24.00 (17.00, 33.00) | **0.002** | 0.518 |
| TB (umol/L) | 14.30 (11.40, 17.90) | 14.70 (11.50, 18.80) | 0.311 |  | 14.80 (11.25, 18.50) | 14.70 (11.30, 18.55) | 0.899 | 0.657 |
| DB (umol/L) | 2.70 (2.10, 3.50) | 2.80 (2.10, 3.40) | 0.604 |  | 2.90 (2.10, 3.50) | 2.70 (2.05, 3.50) | 0.506 | 0.777 |
| IB (umol/L) | 11.50 (9.15, 14.40) | 12.00 (9.10, 15.20) | 0.226 |  | 11.80 (9.20, 15.15) | 11.90 (8.80, 15.20) | 0.876 | 0.542 |
| TP (g/L) | 68.30 (63.60, 72.35) | 70.60 (66.80, 74.00) | **<0.001** |  | 69.00 (64.35, 72.00) | 69.00 (64.45, 73.45) | 0.380 | 0.432 |
| ALB (g/L) | 43.30 (41.10, 45.30) | 43.70 (41.70, 45.40) | 0.112 |  | 43.50 (41.25, 45.50) | 42.60 (40.95, 45.10) | 0.240 | 0.724 |
| GLB (g/L) | 24.90 (22.35, 27.75) | 26.40 (23.80, 30.50) | **<0.001** |  | 24.80 (22.30, 27.85) | 26.10 (23.30, 28.80) | **0.022** | 0.345 |
| ALP (U/L) | 85.00 (69.00, 101.00) | 88.00 (71.00, 107.00) | **0.039** |  | 84.00 (67.50, 102.50) | 89.00 (72.00, 104.50) | 0.278 | 0.690 |
| GGT (U/L) | 23.60 (17.40, 33.05) | 28.10 (20.80, 42.70) | **<0.001** |  | 22.90 (17.40, 33.75) | 28.80 (20.75, 44.40) | **0.002** | 0.772 |
| CHE (ku/L) | 8.26 (7.32, 9.23) | 8.34 (7.48, 9.28) | 0.366 |  | 8.22 (7.48, 9.34) | 8.51 (7.28, 9.52) | 0.952 | 0.504 |
| TBA (μmol/L) | 3.90 (2.50, 5.75) | 4.30 (2.80, 6.40) | **0.014** |  | 4.10 (2.70, 6.45) | 4.30 (2.50, 7.15) | 0.941 | 0.353 |
| LDH (U/L) | 156.00 (138.00, 172.00) | 164.00 (142.00, 184.00) | **0.012** |  | 157.00 (137.00, 177.00) | 158.00 (133.00, 180.45) | 0.916 | 0.617 |
| Renal function tests |  |  |  |  |  |  |  |  |
| BUN (mmol/L) | 5.55 (4.65, 6.34) | 5.38 (4.55, 6.43) | 0.777 |  | 5.15 (4.42, 6.22) | 5.45 (4.61, 6.56) | 0.052 | 0.185 |
| SCr (umol/L) | 65.00 (56.00, 74.00) | 65.00 (56.00, 76.00) | 0.492 |  | 65.00 (58.00, 72.00) | 65.00 (56.50, 73.00) | 0.800 | 0.646 |
| eGFR [ml/(min·1.73m2)] | 98.83 (92.65, 105.34) | 100.41 (93.25, 108.48) | 0.077 |  | 100.40 (93.96, 106.99) | 100.99 (91.02, 108.71) | 0.855 | 0.296 |
| UA (umol/L) | 310.00 (265.50, 362.50) | 338.00 (290.00, 382.00) | **<0.001** |  | 311.00 (266.00, 371.50) | 328.00 (281.50, 371.00) | 0.154 | 0.682 |
| Serum electrolytes |  |  |  |  |  |  |  |  |
| Potassium (mmo/L) | 3.94 (3.72, 4.16) | 3.95 (3.77, 4.16) | 0.719 |  | 3.95 (3.73, 4.14) | 3.87 (3.62, 4.08) | 0.054 | 0.128 |
| Sodium (mmo/L) | 140.00 (138.00, 141.00) | 139.00 (138.00, 141.00) | **0.015** |  | 140.00 (139.00, 141.00) | 139.00 (138.00, 141.00) | **0.004** | 0.444 |
| Chloride (mmo/L) | 105.00 (104.00, 107.00) | 105.00 (103.00, 106.00) | **<0.001** |  | 105.00 (104.00, 107.00) | 105.00 (103.00, 106.00) | **0.002** | 0.729 |
| Calcium (mmo/L) | 2.25 (2.18, 2.32) | 2.28 (2.20, 2.35) | **0.007** |  | 2.26 (2.19, 2.35) | 2.26 (2.16, 2.35) | 0.665 | 0.983 |
| Phosphate (mmo/L) | 1.17 (1.06, 1.29) | 1.17 (1.06, 1.28) | 0.605 |  | 1.19 (1.08, 1.30) | 1.18 (1.06, 1.30) | 0.744 | 0.252 |
| Lipid profile |  |  |  |  |  |  |  |  |
| TC (mmo/L) | 4.41 (3.80, 4.95) | 4.48 (3.80, 5.17) | 0.199 |  | 4.36 (3.70, 4.97) | 4.46 (3.94, 5.26) | 0.138 | 0.854 |
| TG (mmo/L) | 1.35 (0.99, 1.94) | 1.60 (1.14, 2.50) | **<0.001** |  | 1.50 (1.09, 1.98) | 1.83 (1.22, 2.94) | **0.001** | 0.065 |
| HDL (mmo/L) | 1.06 (0.89, 1.23) | 0.99 (0.85, 1.16) | **0.002** |  | 1.04 (0.91, 1.18) | 0.95 (0.84, 1.14) | **0.014** | 0.455 |
| LDL (mmo/L) | 2.80 (2.31, 3.25) | 2.83 (2.37, 3.40) | 0.304 |  | 2.79 (2.26, 3.13) | 2.83 (2.44, 3.42) | 0.118 | 0.643 |
| **Hormones** |  |  |  |  |  |  |  |  |
| T3 (ng/ml) | 0.97 (0.86, 1.11) | 0.96 (0.85, 1.10) | 0.406 |  | 1.00 (0.89, 1.12) | 0.99 (0.83, 1.15) | 0.550 | 0.150 |
| T4 (ug/dL) | 7.43 (6.41, 8.55) | 7.40 (6.30, 8.78) | 0.979 |  | 7.16 (6.26, 8.29) | 7.26 (5.98, 8.35) | 0.917 | 0.113 |
| TSH (uIU/mL) | 2.31 (1.47, 3.41) | 2.37 (1.43, 3.61) | 0.758 |  | 2.64 (1.77, 3.87) | 2.22 (1.32, 3.33) | **0.013** | 0.225 |
| 25(OH)D (nmol L) | 15.93 (10.96, 20.74) | 14.16 (10.85, 18.97) | 0.054 |  | 15.60 (11.36, 19.27) | 14.84 (10.87, 19.42) | 0.614 | 0.989 |
| **Complete blood count** |  |  |  |  |  |  |  |  |
| WBC (10^9/L) | 5.75 (4.94, 6.70) | 6.00 (5.12, 7.25) | **0.004** |  | 5.65 (4.84, 6.56) | 5.65 (4.81, 6.42) | 0.876 | 0.064 |
| RBC (10^12/L) | 4.83 (4.55, 5.15) | 4.90 (4.65, 5.29) | **0.007** |  | 4.84 (4.58, 5.17) | 4.93 (4.61, 5.20) | 0.496 | 0.768 |
| Hb (g/L) | 148.00 (138.50, 158.00) | 153.00 (141.00, 160.00) | **0.004** |  | 150.00 (139.00, 161.00) | 151.00 (140.00, 160.50) | 0.726 | 0.370 |
| Plt (10^9/L) | 180.00 (151.50, 225.00) | 182.00 (148.00, 228.00) | 0.833 |  | 173.00 (144.50, 209.00) | 171.00 (132.00, 204.00) | 0.363 | **<0.001** |
| LYMPH (10^9/L) | 1.90 (1.56, 2.40) | 1.96 (1.54, 2.50) | 0.407 |  | 1.90 (1.54, 2.38) | 2.00 (1.60, 2.31) | 0.343 | 0.756 |
| MONO (10^9/L) | 0.36 (0.30, 0.46) | 0.40 (0.30, 0.50) | **0.038** |  | 0.36 (0.30, 0.41) | 0.35 (0.30, 0.42) | 0.809 | 0.075 |
| NEUT (10^9/L) | 3.20 (2.59, 3.90) | 3.50 (2.79, 4.30) | **<0.001** |  | 3.20 (2.60, 3.81) | 3.10 (2.62, 3.79) | 0.808 | **0.024** |
| EO (10^9/L) | 0.10 (0.08, 0.20) | 0.11 (0.09, 0.20) | 0.112 |  | 0.10 (0.09, 0.18) | 0.10 (0.10, 0.20) | 0.097 | 0.504 |
| BASO (10^9/L) | 0.01 (0.00, 0.02) | 0.01 (0.00, 0.02) | 0.296 |  | 0.01 (0.00, 0.02) | 0.01 (0.00, 0.03) | 0.784 | 0.499 |
| **AIP** | 0.12 (-0.05, 0.30) | 0.21 (0.04, 0.42) | **<0.001** |  | 0.16 (-0.03, 0.34) | 0.27 (0.04, 0.49) | **<0.001** | 0.100 |

*Abbreviation:* DKD, diabetic kidney disease; ES-DKD, early-stage diabetic kidney disease; HTN, hypertension; MASLD, metabolic dysfunction-associated steatotic liver disease; CVD, cardiovascular disease; DR, diabetic retinopathy; DSPN, diabetic sensorimotor polyneuropathy; DPVD, diabetic peripheral vascular disease; FM, family history; DM, diabetes mellitus; OHA, oral hypoglycemic agents; LLA, lipid-lowering agents; Anti-inflamm, anti-inflammatory agents; Renoprot, renoprotective agents; AH, antihypertensive agents; SBP, systolic blood pressure; DBP, diastolic blood pressure; BMI, body mass index; HbA1c, glycated hemoglobin; FBG, fasting blood glucose; FINS, fasting insulin; FCP, fasting C-peptide; 2hBG, 2-hour postprandial blood glucose; 2hINS, 2-hour postprandial insulin; 2hCP, 2-hour postprandial C-peptide; AST, aspartate aminotransferase; ALT, alanine aminotransferase; TB, total bilirubin; DB, direct bilirubin; IB, indirect bilirubin; TP, total protein; ALB, albumin; GLB, globulin; ALP, alkaline phosphatase; GGT, gamma-glutamyl transferase; CHE, cholinesterase; TBA, total bile acids; LDH, lactate dehydrogenase; BUN, blood urea nitrogen; SCr, serum creatinine; eGFR, estimated glomerular filtration rate; UA, uric acid; TC, total cholesterol; TG, triglycerides; HDL, high-density lipoprotein; LDL, low-density lipoprotein; T3, triiodothyronine; T4, thyroxine; TSH, thyroid-stimulating hormone; 25(OH)D, 25-hydroxyvitamin D; WBC, white blood cell; RBC, red blood cell; Hb, hemoglobin; Plt, platelet; LYMPH, lymphocyte; MONO, monocyte; NEUT, neutrophil; EO, eosinophil; BASO, basophil; AIP, atherogenic index of plasma.

**Table S5** Univariate Cox regression analysis for early-stage diabetic kidney disease (ES-DKD).

| characteristics | B | SE | HR | CI | Z | *P* |
| --- | --- | --- | --- | --- | --- | --- |
| Sex-Female | -0.321 | 0.131 | 0.725 | 0.561-0.938 | -2.451 | **0.014** |
| Age | -0.001 | 0.006 | 0.999 | 0.987-1.011 | -0.129 | 0.897 |
| Ethnicity-Others | -0.053 | 0.258 | 0.948 | 0.572-1.572 | -0.207 | 0.836 |
| Career | -0.242 | 0.128 | 0.785 | 0.611-1.010 | -1.885 | 0.059 |
| Marriage | 0.103 | 0.504 | 1.109 | 0.413-2.978 | 0.205 | 0.838 |
| Course | 0.003 | 0.010 | 1.003 | 0.984-1.023 | 0.308 | 0.758 |
| FM of DM | 0.056 | 0.124 | 1.058 | 0.829-1.349 | 0.453 | 0.650 |
| FM of HTN | -0.122 | 0.169 | 0.885 | 0.635-1.233 | -0.722 | 0.470 |
| SBP | 0.011 | 0.004 | 1.011 | 1.004-1.019 | 3.106 | **0.002** |
| DBP | 0.016 | 0.006 | 1.016 | 1.005-1.028 | 2.780 | **0.005** |
| BMI | 0.043 | 0.020 | 1.044 | 1.003-1.087 | 2.126 | **0.033** |
| Smoking | 0.194 | 0.131 | 1.214 | 0.939-1.569 | 1.478 | 0.139 |
| Drinking | 0.040 | 0.146 | 1.041 | 0.782-1.386 | 0.277 | 0.782 |
| Insulins | 0.354 | 0.121 | 1.424 | 1.123-1.805 | 2.920 | **0.003** |
| OHA | 0.277 | 0.581 | 1.319 | 0.423-4.116 | 0.477 | 0.634 |
| LLA | 0.114 | 0.121 | 1.121 | 0.885-1.420 | 0.949 | 0.343 |
| Anti.inflamm | 0.364 | 0.131 | 1.439 | 1.112-1.862 | 2.768 | **0.006** |
| Renoprot | 0.458 | 0.324 | 1.580 | 0.838-2.980 | 1.414 | 0.157 |
| AH | 0.204 | 0.127 | 1.226 | 0.956-1.573 | 1.603 | 0.109 |
| FBG | 0.099 | 0.018 | 1.104 | 1.067-1.143 | 5.622 | **<0.001** |
| FINS | 0.007 | 0.008 | 1.007 | 0.991-1.023 | 0.892 | 0.372 |
| FCP | 0.194 | 0.089 | 1.214 | 1.019-1.446 | 2.174 | **0.030** |
| 2hBG | 0.041 | 0.012 | 1.041 | 1.018-1.066 | 3.443 | **<0.001** |
| 2hINS | -0.001 | 0.002 | 0.999 | 0.994-1.003 | -0.652 | 0.515 |
| 2hCP | -0.063 | 0.028 | 0.939 | 0.888-0.993 | -2.204 | **0.028** |
| AST | 0.012 | 0.003 | 1.012 | 1.007-1.018 | 4.409 | **<0.001** |
| ALT | 0.011 | 0.003 | 1.011 | 1.006-1.017 | 3.874 | **<0.001** |
| TB | 0.001 | 0.009 | 1.001 | 0.984-1.019 | 0.137 | 0.891 |
| DB | -0.015 | 0.026 | 0.985 | 0.936-1.037 | -0.579 | 0.563 |
| IB | 0.008 | 0.013 | 1.008 | 0.983-1.034 | 0.640 | 0.522 |
| TP | 0.043 | 0.010 | 1.044 | 1.023-1.065 | 4.208 | **<0.001** |
| ALB | 0.008 | 0.018 | 1.008 | 0.973-1.045 | 0.458 | 0.647 |
| GLB | 0.068 | 0.013 | 1.070 | 1.043-1.099 | 5.115 | **<0.001** |
| ALP | 0.003 | 0.002 | 1.003 | 0.999-1.008 | 1.544 | 0.123 |
| GGT | 0.006 | 0.001 | 1.006 | 1.004-1.009 | 4.981 | **<0.001** |
| CHE | 0.046 | 0.043 | 1.047 | 0.963-1.138 | 1.074 | 0.283 |
| TBA | 0.013 | 0.014 | 1.013 | 0.986-1.040 | 0.930 | 0.353 |
| BUN | 0.024 | 0.013 | 1.024 | 0.999-1.050 | 1.865 | 0.062 |
| Cr | 0.005 | 0.005 | 1.005 | 0.996-1.014 | 1.032 | 0.302 |
| eGFR | 0.002 | 0.005 | 1.002 | 0.993-1.011 | 0.402 | 0.688 |
| UA | 0.002 | 0.001 | 1.002 | 1.001-1.004 | 2.762 | **0.006** |
| Potassium | -0.039 | 0.183 | 0.962 | 0.673-1.377 | -0.211 | 0.833 |
| Sodium | -0.061 | 0.028 | 0.940 | 0.891-0.993 | -2.218 | **0.027** |
| Chloride | -0.086 | 0.023 | 0.917 | 0.877-0.959 | -3.831 | **<0.001** |
| Calcium | 0.902 | 0.537 | 2.465 | 0.861-7.057 | 1.681 | 0.093 |
| Phosphate | -0.543 | 0.349 | 0.581 | 0.293-1.152 | -1.556 | 0.120 |
| AIP | 0.742 | 0.190 | 2.100 | 1.448-3.046 | 3.910 | **<0.001** |
| TC | 0.032 | 0.064 | 1.032 | 0.911-1.169 | 0.497 | 0.619 |
| TG | 0.107 | 0.031 | 1.113 | 1.046-1.184 | 3.399 | **<0.001** |
| HDL | -0.597 | 0.261 | 0.550 | 0.330-0.918 | -2.290 | **0.022** |
| LDL | 0.109 | 0.089 | 1.116 | 0.937-1.327 | 1.232 | 0.218 |
| LDH | 0.007 | 0.002 | 1.007 | 1.003-1.011 | 3.295 | **<0.001** |
| 25(OH)D | -0.015 | 0.008 | 0.986 | 0.971-1.000 | -1.934 | 0.053 |
| T3 | -0.301 | 0.257 | 0.740 | 0.447-1.225 | -1.172 | 0.241 |
| T4 | 0.047 | 0.031 | 1.048 | 0.985-1.115 | 1.490 | 0.136 |
| TSH | -0.002 | 0.002 | 0.998 | 0.993-1.002 | -1.133 | 0.257 |
| WBC | 0.095 | 0.039 | 1.100 | 1.019-1.187 | 2.456 | **0.014** |
| RBC | 0.367 | 0.134 | 1.443 | 1.111-1.875 | 2.748 | **0.006** |
| Hb | 0.014 | 0.004 | 1.014 | 1.006-1.022 | 3.295 | **<0.001** |
| Plt | 0.000 | 0.001 | 1.000 | 0.998-1.002 | 0.098 | 0.922 |
| LYMPH | 0.008 | 0.091 | 1.008 | 0.844-1.204 | 0.088 | 0.930 |
| MONO | -0.081 | 0.184 | 0.923 | 0.644-1.322 | -0.439 | 0.661 |
| NEUT | 0.049 | 0.019 | 1.050 | 1.011-1.091 | 2.518 | **0.012** |
| EO | -0.032 | 0.230 | 0.968 | 0.617-1.519 | -0.140 | 0.889 |
| BASO | 0.873 | 0.821 | 2.394 | 0.479-11.965 | 1.063 | 0.288 |
| HbA1c | 0.147 | 0.028 | 1.159 | 1.098-1.223 | 5.329 | **<0.001** |
| CVD | 0.030 | 0.124 | 1.030 | 0.808-1.314 | 0.240 | 0.810 |
| HTN | 0.150 | 0.128 | 1.162 | 0.905-1.492 | 1.177 | 0.239 |
| DSPN | -0.036 | 0.154 | 0.965 | 0.713-1.305 | -0.231 | 0.817 |
| DR | -0.037 | 0.121 | 0.964 | 0.761-1.221 | -0.307 | 0.759 |
| DPVD | 0.335 | 0.140 | 1.397 | 1.063-1.838 | 2.394 | **0.017** |
| MASLD | 0.123 | 0.121 | 1.131 | 0.893-1.433 | 1.020 | 0.308 |

*Abbreviation*: SE, standard error; HR, hazard ratio; CI, confidence interval; FM, family history; DM, diabetes mellitus; HTN, hypertension; OHA, oral hypoglycemic agents; LLA, lipid-lowering agents; Anti-inflamm, anti-inflammatory agents; Renoprot, renoprotective agents; AH, antihypertensive agents; SBP, systolic blood pressure; DBP, diastolic blood pressure; BMI, body mass index; FBG, fasting blood glucose; FINS, fasting insulin; FCP, fasting C-peptide; 2hBG, 2-hour postprandial blood glucose; 2hINS, 2-hour postprandial insulin; 2hCP, 2-hour postprandial C-peptide; AST, aspartate aminotransferase; ALT, alanine aminotransferase; TB, total bilirubin; DB, direct bilirubin; IB, indirect bilirubin; TP, total protein; ALB, albumin; GLB, globulin; ALP, alkaline phosphatase; GGT, gamma-glutamyl transferase; CHE, cholinesterase; TBA, total bile acids; LDH, lactate dehydrogenase; BUN, blood urea nitrogen; Cr, creatinine; eGFR, estimated glomerular filtration rate; UA, uric acid; TC, total cholesterol; TG, triglycerides; HDL, high-density lipoprotein; LDL, low-density lipoprotein; 25(OH)D, 25-hydroxyvitamin D; T3, triiodothyronine; T4, thyroxine; TSH, thyroid-stimulating hormone; WBC, white blood cell; RBC, red blood cell; Hb, hemoglobin; Plt, platelet; LYMPH, lymphocyte; MONO, monocyte; NEUT, neutrophil; EO, eosinophil; BASO, basophil; HbA1c, glycated hemoglobin; CVD, cardiovascular disease; HTN, hypertension; DSPN, diabetic sensorimotor polyneuropathy; DR, diabetic retinopathy; DPVD, diabetic peripheral vascular disease; MASLD, metabolic dysfunction-associated steatotic liver disease; AIP, atherogenic index of plasma.

**Table S6** Multivariable Cox regression analysis of the final selected predictors using backward stepwise selection.

| characteristics | B | SE | HR | CI | Z | P | Method |
| --- | --- | --- | --- | --- | --- | --- | --- |
| Anti.inflamm | 0.356 | 0.134 | 1.427 | 1.098-1.856 | 2.658 | 0.008 | backward |
| Sex | -0.531 | 0.141 | 0.588 | 0.446-0.776 | -3.753 | <0.001 | backward |
| AIP | 0.487 | 0.207 | 1.628 | 1.084-2.444 | 2.349 | 0.019 | backward |
| UA | 0.001 | 0.001 | 1.001 | 1.000-1.003 | 1.557 | 0.119 | backward |
| GLB | 0.05 | 0.014 | 1.051 | 1.022-1.082 | 3.457 | <0.001 | backward |
| HbA1c | 0.164 | 0.03 | 1.178 | 1.110-1.251 | 5.374 | <0.001 | backward |
| LDH | 0.007 | 0.002 | 1.007 | 1.002-1.011 | 3.014 | 0.003 | backward |
| GGT | 0.003 | 0.001 | 1.003 | 1.001-1.006 | 2.438 | 0.015 | backward |
| SBP | 0.007 | 0.004 | 1.007 | 0.999-1.014 | 1.664 | 0.096 | backward |

*Abbreviation*: SE, standard error; HR, hazard ratio; CI, confidence interval; Anti-inflamm, anti-inflammatory agents; AIP, atherogenic index of plasma; UA, uric acid; GLB, globulin; HbA1c, glycated hemoglobin; LDH, lactate dehydrogenase; GGT, gamma-glutamyl transferase; SBP, systolic blood pressure.
