# Supplementary material for: Prognostic value of the atherogenic index of plasma for early-stage diabetic kidney disease in type 2 diabetes: a retrospective cohort study using supervised machine learning
Source: Front Nutr. 2026 Jun 30;13:1844510. doi: 10.3389/fnut.2026.1844510 (PMC13377558; doi:10.3389/fnut.2026.1844510)
Supplement: Supplementary file 2 [file Supplementary_file_2.DOCX]

##################################################

#1. Survival Random Forest

##################################################

library(survex) # SHAP for survival analysis

library(randomForestSRC) # Random forest for survival analysis

rsfx = reactive({

# observeEvent(input$runrsf, {

req(x_se_rsf())

df = splitdata()[[1]] %>% select(y_se_rsf(), timersf(), x_se_rsf()) %>%

dplyr::rename("status" = 1, "time" = 2)

set.seed(123)

AA = tune.nodesize(Surv(time, status) ~ ., df)

AA$nsize.opt

# Random Survival Forest 1

set.seed(123)

rf.model <- rfsrc(Surv(time, status) ~ ., data = df,

ntree = 1000,

splitrule = "logrank",

importance = TRUE,

nodesize = AA$nsize.opt)

return(rf.model)

# })

})

##########################################################

#2. Coxboost

##########################################################

Ml_cox_boost <- function(dev = mydata, y = "a", ti = "t", xva = "xa") {

dfcox = dev %>% dplyr::select(y, ti, xva) %>%

dplyr::rename("y" = 1, "time" = 2) %>% na.omit()

# dfglm2 = vad %>% dplyr::select(y, ti, xva) %>%

# dplyr::rename("y" = 1, "time" = 2)

# devData <- na.omit(devData)

library(caret)

# Perform dummy variable encoding. Note: Y should not be pre-set as a factor,

# otherwise it will be converted into dummy variables!

dvfunc <- dummyVars(~., data = dfcox, fullRank = T)

df2 <- predict(dvfunc, newdata = dfcox) %>% as.data.frame()

# x <- dfcox %>% select(-1, -2)

x <- df2 %>% select(-1, -2)

train.x <- as.matrix(x)

time <- dfcox$time

status <- dfcox$y

library(CoxBoost)

## 1. Find optimal number of boosting steps

cv.res <- cv.CoxBoost(time = time, status = status, x = train.x, maxstepno = 1000,

K = 10, type = "verweij", penalty = 100)

opstep <- cv.res$optimal.step

## 2. Output optimal penalty

optim.res <- optimCoxBoostPenalty(time = time, status = status, x = train.x,

trace = FALSE, start.penalty = 500)

oppenalty <- optim.res$penalty

# 3. Fit the optimal model

cbfit <- CoxBoost(time = time, status = status, x = train.x,

stepno = cv.res$optimal.step,

penalty = optim.res$penalty)

# summary(cbfit)

return(list(opstep = opstep, oppenalty = oppenalty, cbfit = cbfit))

}

coxresult <- reactive({

dfuil = splitdata()[[1]] %>% select(y_se_cox(), timevarcox(), x_se_cox())

xa = Ml_cox_boost(dev = dfuil, y = y_se_cox(), ti = timevarcox(), xva = x_se_cox())

return(xa)

})

*##################################################*

#3. Lasso_COX

*##################################################*

lasscox_dumy <- function(dta = dev, dta2 = vad, y = y, ti = ti, x = c("age"),

nfolds = nfolds, lassoran = lassoran, alpha = alpha) {

dfx = dta %>% dplyr::select(y, ti, x) %>% na.omit() %>%

dplyr::rename("status" = 1, "time" = 2) *# %>%*

*# dplyr::filter(!time == 0)*

dfx$time[dfx$time <= 0] = 0.003

dfx2 = dta2 %>% dplyr::select(y, ti, x) %>% na.omit() %>%

dplyr::rename("status" = 1, "time" = 2) *# %>%*

*# dplyr::filter(!time == 0)*

dfx2$time[dfx2$time <= 0] = 0.003

library(caret)

*# Perform dummy variable encoding. Note: Y should not be pre-set as a factor,*

*# otherwise it will be converted into dummy variables!*

dvfunc <- dummyVars(~., data = dfx, fullRank = T)

df2 <- predict(dvfunc, newdata = dfx) %>% as.data.frame()

df3 <- predict(dvfunc, newdata = dfx2) %>% as.data.frame()

yy <- as.matrix(Surv(df2[, 2], df2[, 1]))

xx <- as.matrix(as.data.frame(df2[, -c(1, 2)]))

fit <- glmnet(xx, yy, family = "cox", alpha = alpha)

*#*

set.seed(lassoran)

cv.fit <- cv.glmnet(xx, yy, family = "cox", nfolds = nfolds)

*# plot(cv.fit)*

*# abline(v = log(c(cv.fit$lambda.min, cv.fit$lambda.1se)), lty = 2, lwd = 1.5)*

*# If using the minimum value*

min = cv.fit$lambda.min

Coefficients <- coef(fit, s = cv.fit$lambda.min)

Active.Index <- which(Coefficients != 0)

Active.Coefficients <- Coefficients[Active.Index]

Active.Index

ac1 = Active.Coefficients

out1 = row.names(Coefficients)[Active.Index]

*# If using the 1-standard error value*

se = cv.fit$lambda.1se

Coefficients <- coef(fit, s = cv.fit$lambda.1se)

Active.Index <- which(Coefficients != 0)

Active.Coefficients <- Coefficients[Active.Index]

Active.Index

ac2 = Active.Coefficients

out2 = row.names(Coefficients)[Active.Index]

ti = paste0("coxph(Surv(time, status) ~ ", paste(out1, collapse = " + "), ", data = df2)")

Lasso_Cox = eval(parse(text = ti))

*# ti = paste0("coxph(Surv(time, status) ~ ", paste(out2, collapse = " + "), ", data = df2")*

train_Lasso_Cox = data.frame(df2[, c("time", "status")], predicted = predict(Lasso_Cox, type = 'risk', newdata = df2))

test_Lasso_Cox = data.frame(df3[, c("time", "status")], predicted = predict(Lasso_Cox, type = 'risk', newdata = df3))

return(list(fit1 = fit, fit2 = cv.fit,

out1 = out1, out2 = out2,

ac1 = ac1, ac2 = ac2,

min = min, se = se,

train_Lasso_Cox = train_Lasso_Cox,

test_Lasso_Cox = test_Lasso_Cox

))

}

Latrain_table0 <- reactive({

xa = lasscox_dumy(dta = splitdata()[[1]], dta2 = splitdata()[[2]],

y = y_se_lacox(), ti = timevarcox(), x = x_se_lacox(),

input$cvlasso, lassoran = input$lassorandom, alpha = input$glmtype)

xaa = xa[[9]] %>% rename("Lasso_cox" = 3)

return(xaa)

})

########################################################

#4. XGBoost

########################################################

xgb0 <- reactive({

dfx = splitdata()[[1]] %>% select(y_se_xgb(), timevarxgb0(), x_se_xgb()) %>%

dplyr::rename("status" = 1, "time" = 2)

library(caret)

# Perform dummy variable encoding. Note: Y should not be pre-set as a factor,

# otherwise it will be converted into dummy variables!

dvfunc <- dummyVars(~., data = dfx, fullRank = T)

df <- predict(dvfunc, newdata = dfx) %>% as.data.frame()

set.seed(123)

xgb_param = list(objective = "survival:cox",

booster = "gbtree",

eval_metric = "cox-nloglik",

eta = 0.03,

max_depth = 3,

subsample = 1,

colsample_bytree = 1,

gamma = 0.5)

traindata_y = ifelse(df$status == 1, df$time, -df$time)

library(xgboost)

traindata_matrix = xgb.DMatrix(data = as.matrix(df[, 3:ncol(df)]), label = traindata_y)

xgboost = xgb.train(params = xgb_param, data = traindata_matrix, nrounds = 100,

watchlist = list(val2 = traindata_matrix),

early_stopping_rounds = 50)

return(xgboost)

})

xgbtrain_table0 <- reactive({

req(x_se_xgb())

dfx = splitdata()[[1]] %>% select(y_se_xgb(), timevarxgb0(), x_se_xgb()) %>%

dplyr::rename("status" = 1, "time" = 2)

library(caret)

# Perform dummy variable encoding. Note: Y should not be pre-set as a factor,

# otherwise it will be converted into dummy variables!

dvfunc <- dummyVars(~., data = dfx, fullRank = T)

df <- predict(dvfunc, newdata = dfx) %>% as.data.frame()

train_xgb_P = data.frame(df[, c("time", "status")],

predicted = as.numeric(predict(xgb0(), newdata = as.matrix(df[, 3:ncol(df)]), type = "risk")))

train_xgb_P = train_xgb_P %>% rename("xgb" = 3)

return(train_xgb_P)

})

########################################################

#5. Superpc

########################################################

spctrain_table0 <- reactive({

dfx = splitdata()[[1]] %>% select(y_se_spc(), timevarspc0(), x_se_spc()) %>%

dplyr::rename("status" = 1, "time" = 2)

library(caret)

*# Perform dummy variable encoding. Note: Y should not be pre-set as a factor,*

*# otherwise it will be converted into dummy variables!*

dvfunc <- dummyVars(~., data = dfx, fullRank = T)

df <- predict(dvfunc, newdata = dfx) %>% as.data.frame()

set.seed(123)

traindata_superpc = list(x = t(df[, -c(1, 2)]), y = df$time,

censoring.status = df$status,

featurenames = colnames(df)[-c(1, 2)])

superpc_fit = superpc.train(data = traindata_superpc, type = 'survival', s0.perc = 0.5) *# default*

superpc = superpc.cv(superpc_fit, traindata_superpc, n.threshold = 20, *# default*

n.fold = 10,

n.components = 3,

min.features = 5,

max.features = nrow(traindata_superpc$x),

compute.fullcv = TRUE,

compute.preval = TRUE)

superpc_ff_train = superpc.predict(superpc_fit, traindata_superpc, traindata_superpc,

threshold = superpc$thresholds[which.max(superpc[["scor"]][1, ])],

n.components = 1)

train_superpc = data.frame(df[, c("time", "status")],

predicted = as.numeric(superpc_ff_train$v.pred))

train_superpc = train_superpc %>% rename("Superpc" = 3)

return(train_superpc)

})

########################################################

#6. plsRcox

########################################################

library(plsRcox)

# Output validation set predicted probabilities

pls <- reactive({

dfx = splitdata()[[1]] %>% select(y_se_pls(), timevarpls0(), x_se_pls()) %>%

dplyr::rename("status" = 1, "time" = 2)

set.seed(123)

library(caret)

# Perform dummy variable encoding. Note: Y should not be pre-set as a factor,

# otherwise it will be converted into dummy variables!

dvfunc <- dummyVars(~., data = dfx, fullRank = T)

df <- predict(dvfunc, newdata = dfx) %>% as.data.frame()

cv.plsRcox.res = cv.plsRcox(list(x = df[, 3:ncol(df)], time = df$time, status = df$status),

nt = 10, nfold = 10, verbose = F)

plsRcox = plsRcox(df[, 3:ncol(df)], time = df$time, event = df$status, nt = 3)

return(plsRcox)

})

plstrain_table0 <- reactive({

dfx = splitdata()[[1]] %>% select(y_se_pls(), timevarpls0(), x_se_pls()) %>%

dplyr::rename("status" = 1, "time" = 2)

library(caret)

# Perform dummy variable encoding. Note: Y should not be pre-set as a factor,

# otherwise it will be converted into dummy variables!

dvfunc <- dummyVars(~., data = dfx, fullRank = T)

df <- predict(dvfunc, newdata = dfx) %>% as.data.frame()

train_plsRcox = data.frame(df[, c("time", "status")],

predicted = as.numeric(predict(pls(), newdata = as.matrix(df[, 3:ncol(df)]), type = "risk")))

train_plsRcox = train_plsRcox %>% rename("plsRcox" = 3)

return(train_plsRcox)

})
